# Rasa3 controls turnover of endothelial cell adhesion and vascular lumen integrity by a Rap1-dependent mechanism

**DOI:** 10.1371/journal.pgen.1007195

**Published:** 2018-01-30

**Authors:** Patricia Molina-Ortiz, Tanguy Orban, Maud Martin, Audrey Habets, Franck Dequiedt, Stéphane Schurmans

**Affiliations:** 1 Laboratory of Functional Genetics, GIGA-Molecular Biology of Disease, University of Liège, Liège, Belgium; 2 Laboratory of Protein signaling and Interactions Signalisation, GIGA-Molecular Biology of Diseases, University of Liège, Liège, Belgium; Stanford University School of Medicine, UNITED STATES

## Abstract

Rasa3 is a GTPase activating protein of the GAP1 family which targets R-Ras and Rap1. Although catalytic inactivation or deletion of Rasa3 in mice leads to severe hemorrhages and embryonic lethality, the biological function and cellular location of Rasa3 underlying these defects remains unknown. Here, using a combination of loss of function studies in mouse and zebrafish as well as *in vitro* cell biology approaches, we identify a key role for Rasa3 in endothelial cells and vascular lumen integrity. Specific ablation of Rasa3 in the mouse endothelium, but not in megakaryocytes and platelets, lead to embryonic bleeding and death at mid-gestation, recapitulating the phenotype observed in full *Rasa3* knock-out mice. Reduced plexus/sprouts formation and vascular lumenization defects were observed when Rasa3 was specifically inactivated in mouse endothelial cells at the postnatal or adult stages. Similar results were obtained in zebrafish after decreasing Rasa3 expression. *In vitro*, depletion of Rasa3 in cultured endothelial cells increased β1 integrin activation and cell adhesion to extracellular matrix components, decreased cell migration and blocked tubulogenesis. During migration, these Rasa3-depleted cells exhibited larger and more mature adhesions resulting from a perturbed dynamics of adhesion assembly and disassembly which significantly increased their life time. These defects were due to a hyperactivation of the Rap1 GTPase and blockade of FAK/Src signaling. Finally, Rasa3-depleted cells showed reduced turnover of VE-cadherin-based adhesions resulting in more stable endothelial cell-cell adhesion and decreased endothelial permeability. Altogether, our results indicate that Rasa3 is a critical regulator of Rap1 in endothelial cells which controls adhesions properties and vascular lumen integrity; its specific endothelial cell inactivation results in occluded blood vessels, hemorrhages and early embryonic death in mouse, mimicking thus the *Rasa*3^-/-^ mouse phenotype.

## Author summary

Because it delivers oxygen and nutriments to every tissue in the body, the vascular system is essential to vertebrate life. Blood vessels consist of a layer of interconnected endothelial cells delineating a luminal space through which blood flows. Formation of vascular lumens is a critical step in vascular development, as vessels should allow unrestricted blood flow while absorbing the pressure from cardiac activity yet retaining flexibility to adapt to homeostatic needs. Our current knowledge of how lumens are established and maintained is still modest and has come essentially from *in vitro* systems. Here, using a combination of loss of function studies in mouse and zebrafish and *in vitro* cell biology approaches, we show that Rasa3, a GTPase activating protein of the GAP1 family, controls Rap1 activation, endothelial cell adhesion and migration as well as formation of vascular lumens. We also found that inactivation of Rasa3 specifically in mouse endothelial cells lead to embryonic bleeding and death at mid-gestation, recapitulating the phenotype observed in full *Rasa3* knock-out mice.

## Introduction

Blood vessels consist of a layer of interconnected endothelial cells (ECs) delineating a luminal space through which blood flows. Our current knowledge of how lumens are established and maintained is still modest and has come essentially from *in vitro* systems. Only recently, studies have investigated vascular lumen formation *in vivo*: adhesion to surrounding extracellular matrix (ECM), remodeling of EC-EC junctions and actin cytoskeleton-driven cell shape changes are common themes in this complex process [1]. Through loss-of-function experiments, these studies also identified several molecular regulators crucial for lumenogenesis, including cell surface and polarity proteins, kinases and phosphatase, actin interactors and regulators, EC-ECM adhesion proteins and small GTPase signaling components [2–11].

The small GTPase superfamily, which includes the Ras, Rho, Ran, Rab and Arf families, is composed of proteins that act as molecular switches in important signaling pathways. These pathways, which relate to cell proliferation and survival, cell-matrix and cell-cell adhesion, and cytoskeleton dynamics are critical for normal development and physiology and, when deregulated, cause severe life-threatening syndromes and pathologies. Small GTPases activity is controlled by the antagonistic actions of activating guanine exchange factors (GEFs) and repressing GTPase-activating proteins (GAPs). Rasa3 (GAP1IP4BP, R-Ras GAP) is a member of the GAP1 subfamily of Ras GAPs and is known to function as a dual GAP for Rap1 and R-Ras small GTPases [12,13]. While R-Ras has been extensively studied due to its involvement in cancer, Rap1 has recently attracted a lot of attention due to its central role in development and morphogenesis of higher organisms, especially in the cardiovasculature [14].

Mouse models have confirmed a critical role for Rasa3 during development and differentiation. Mice homozygous for the *scat* (severe combined anemia and thrombocytopenia) mutation in the *Rasa3* gene exhibit successive episodes of severe bleeding associated with embryonic and postnatal mortality [15]. Massive hemorrhages are also observed in *Rasa3*^-/-^ embryos expressing a catalytically inactive Rasa3 protein and are associated with death at embryonic day (E) 12.5 to E13.5 [16]. We and others reported that loss-of-function of Rasa3 was associated with a severe thrombocytopenia, providing a possible explanation for the embryonic hemorrhages and lethality [15,17,18]. The thrombocytopenic syndrome was attributed to hyperactivation of Rap1-dependent signaling upstream of αIIbβ3 integrin stimulation, resulting in defects during megakaryopoiesis and/or circulating platelet activation [17,18]. However, compared to full *Rasa3*^*-/-*^ mice, the hemorrhagic phenotype and embryonic lethality were much less severe in mice in which Rasa3 was deleted specifically in the megakaryocyte lineage, suggesting that they might be caused by defects in a different cell type [18]. Here, we tested the hypothesis that embryonic bleeding and lethality associated with *Rasa3* inactivation relate to its important function in endothelial cells and vascular development. We report that mice with endothelial-specific deletion of Rasa3 exhibited severe hemorrhages and embryonic death, recapitulating the *Rasa3*^-/-^ mouse phenotype. By contrast, Rasa3 inactivation specifically in megakaryocytes caused a severe thrombocytopenia but no embryonic lethality. We also show that lack of Rasa3 in ECs is associated with hyperactivation of Rap1 GTPase signaling, deregulation of EC-ECM and EC-EC adhesions and of endothelial tube morphogenesis. Our study thus identifies Rasa3 as a critical regulator of Rap1 activity, adhesion processes and tubulogenesis in ECs.

## Results

### Rasa3 inactivation in EC results in severe bleeding and embryonic death

**Fig 1:**
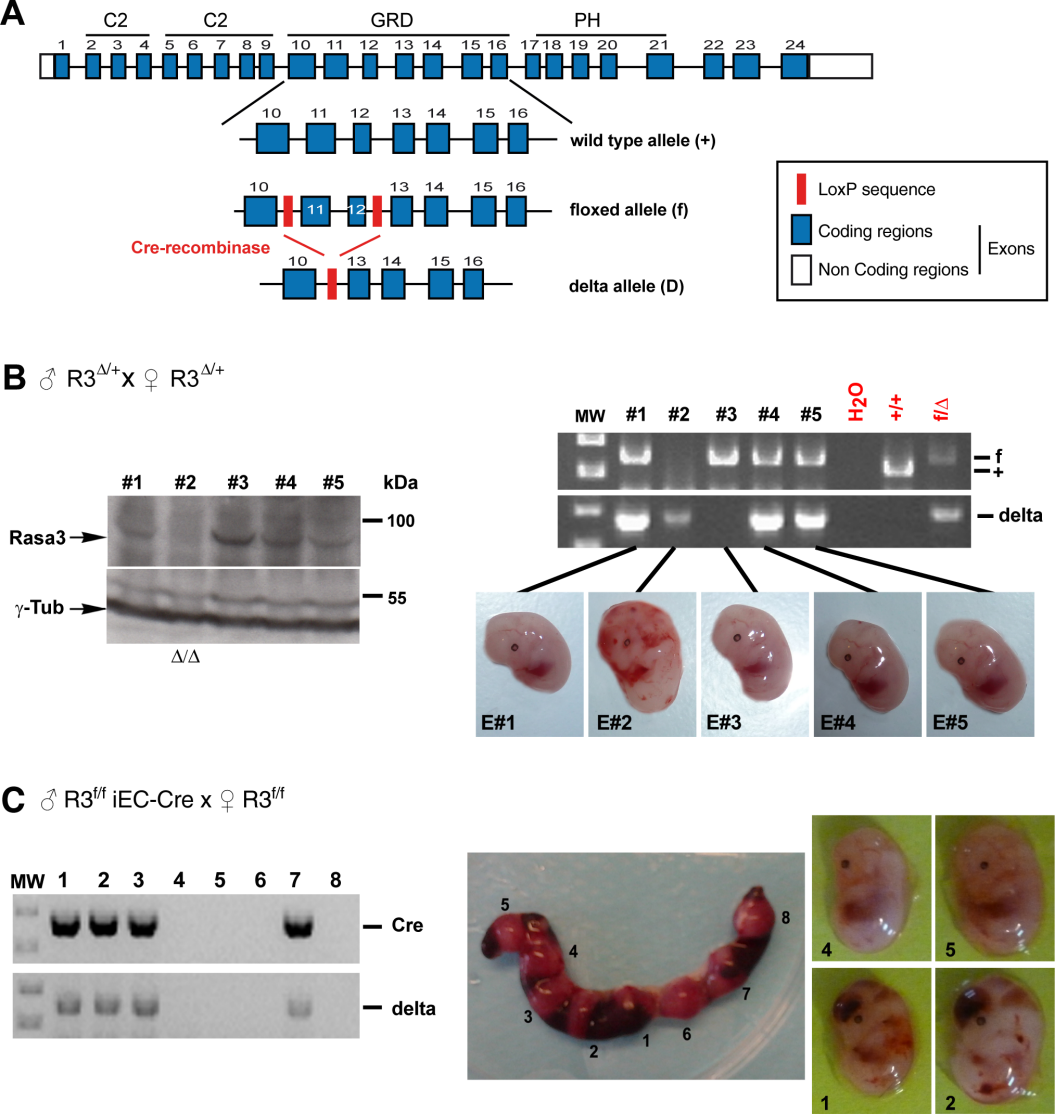
Whole embryo or endothelial-specific inactivation of Rasa3 induces severe bleeding and lethality in E12.5 embryos. **A.** The mouse *Rasa3* gene structure (boxes denote exons, and exons in blue indicate the coding regions) with the corresponding protein domains, C2 (C2), the GAP-related domain (GRD) and the pleckstrin homology domain (PH), are represented. LoxP site insertions in the floxed (f) allele are indicated (red box). The post-recombination delta (∆) allele is represented. **B.** (Left) Immunodetection of Rasa3 and γ-Tubulin by Western blotting on extracts isolated from 5 E12.5 embryos from an *R3*^∆/+^ mice intercross. (Right) Genotyping of embryos described at the left by PCR amplification of the genomic region between the LoxP sites (f) or of the delta *Rasa3* allele. E2 embryo is *R3*^∆/∆^, E1, E4 and E5 are *R3*^∆/+^ embryos and E3 is the *R3*^f/f^ embryo (E3). Results are representative of 5 separate experiments. **C.** Uterine horns of a *R3*^f/f^ female at E12.5 after crossing with a *R3*^f/f^ iEC-Cre male, and ip injected with tamoxifen (center). Eight embryos were genotyped for the Cre transgene and the *R3*^∆^ allele (left). Only embryos positive for the Cre transgene have the *R3*^∆^ allele (E1, E2, E3 and E7) and present severe bleeding (right). Results are representative of 10 separate experiments.

In order to explore the functions of Rasa3 in ECs *in vivo*, we generated a *Rasa3*^flox/+^ (*R3*^f/+^) mutant mouse in which two intronic LoxP sites were introduced upstream of exon 11 and downstream of exon 12 in the *Rasa3* gene (Fig 1A). Exons 11 and 12 of the Rasa3 gene were specifically targeted, as previously described by Iwashita et al. [16]. Deletion of these two exons should lead to the production of a 88 amino acids-truncated catalytically inactive Rasa3 protein, if stable. Doing so, we were sure to inactivate the Rasa3 gene and to reproduce the embryonic lethality of *Rasa3*^*-/-*^ mice. Crossing *R3*^f/+^ with *PGK-Cre* mice generated *R3*^∆/+^ mice with a full body heterozygous deletion of exons 11 and 12, which encodes residues 315–402 of the RASA3 GAP domain. Intercrosses between *R3*^∆/+^ mice failed to yield any live *R3*^∆/∆^ newborns (S1 Table). At E12.5, all *R3*^∆/∆^ embryos displayed widespread hemorrhages, indicative of abnormalities in the vascular or coagulation systems (Fig 1B). When analyzed by Western blot, *R3*^∆/∆^ embryos revealed that homozygous deletion of exon 11 and 12 of the *Rasa3* gene resulted in the absence of the Rasa3 protein (Fig 1B). Since in our hands deletion of Rasa3 specifically in megakaryocytes and platelets was not associated with embryonic lethality or hemorrhages (S1 Table and S1 Fig), we investigated whether this phenotype is observed when Rasa3 is inactivated in ECs. We generated *R3*^f/+^ *Cdh5(PAC)-*CreERT2 (*R3*^f/+^ iEC-Cre) mice by crossing *R3*^f/f^ mice with mice expressing the Cre recombinase under the control of a tamoxifen-inducible, EC-specific promoter. *R3*^f/f^ iEC-Cre mice were obtained by intercrossing *R3*^f/+^ iEC-Cre mice. To achieve EC-specific homozygous inactivation of Rasa3, female *R3*^f/f^ mice were bred with male *R3*^f/f^ iEC-Cre mice. Intraperitoneal (ip) injections of tamoxifen in pregnant female *R3*^f/f^ mice at E8.5, E9.5 and E10.5 correlated with embryonic lethality and absence of Rasa3 mutant newborns (S1 Table). Genotyping of E12.5 embryos revealed deletion of Rasa3 exons 11 and 12 in embryos that were positive for the Cre recombinase (Fig 1C). As was previously observed with the *R3*^∆/∆^ deletion, the lethal phenotype resulting from EC-specific deletion of Rasa3 was 100% penetrant and consistently associated with massive bleeding (S1 Table and Fig 1C). In contrast, sibling *R3*^f/f^ embryos lacking the Cre recombinase transgene did not show any vascular defects.

### EC specific inactivation of Rasa3 induces blood vessel lumenization defects *in vivo*

**Fig 2:**
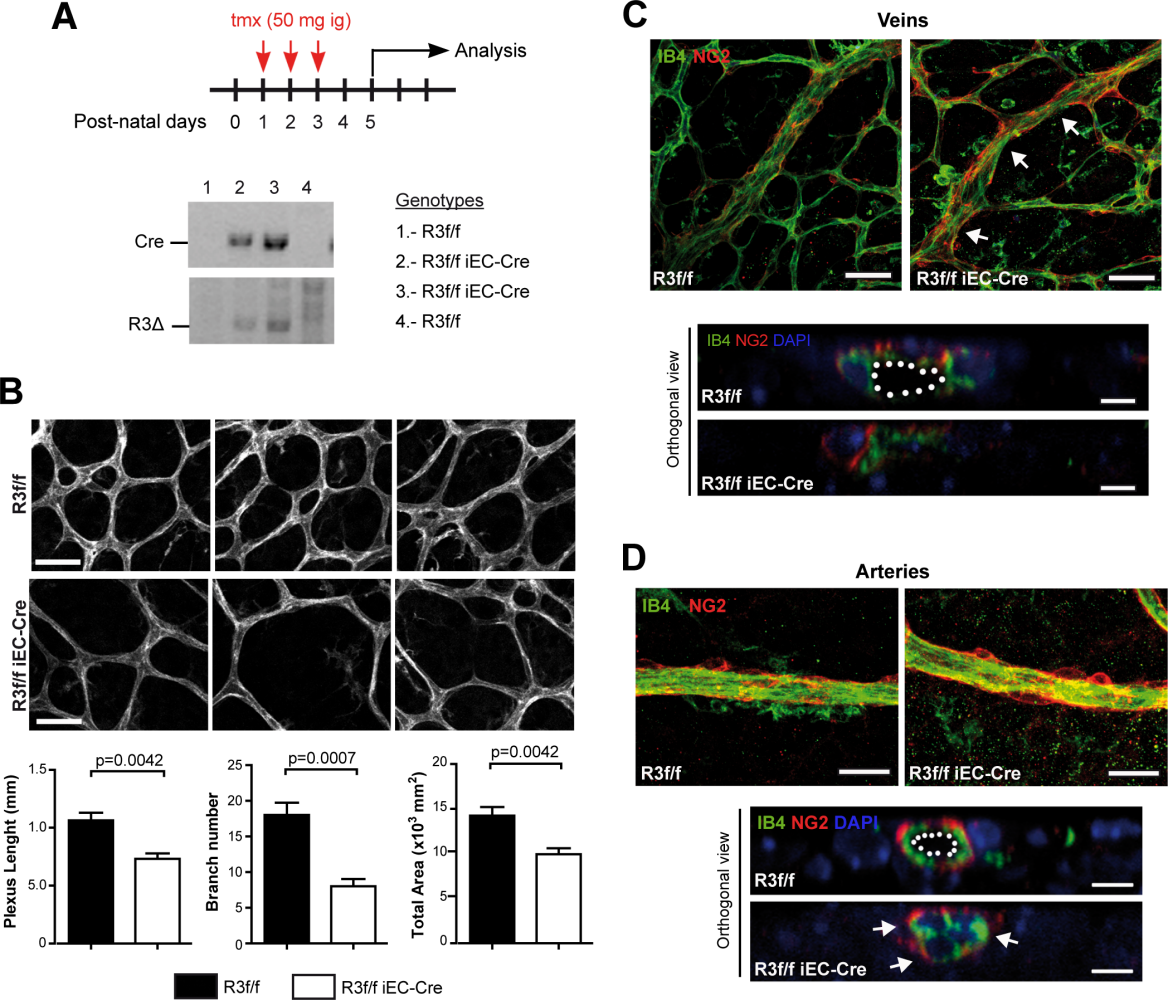
Endothelial deletion of Rasa3 results in defects in retinal vascularization. **A.** Experimental design of deletion of Rasa3 in *R3*^f/f^ and *R3*^f/f^ iEC-Cre pups via tamoxifen (tmx) intragastric (ig) injections at P1, P2 and P3. At P5, the *R3*^∆^ allele was only detected in Cre-positive *R3*^f/f^ newborns. Lower panels: genotyping of 4 mice by PCR for the Cre transgene (above) and the Rasa3^∆^ allele (below) detection. When the Cre transgene is present, the Rasa3^∆^ allele appears, indicating the deletion of exons 11 and 12 of the Rasa3 gene. The genotype of the 4 mice is indicated on the right. **B.** Immunofluorescence analysis of *R3*^f/f^ and *R3*^f/f^ iEC-Cre retinal plexus stained for the IB4 endothelial cell marker (upper images). Representative images of 4 independent experiments are shown. Bars = 50 μm. (Graphs) Quantification of cumulative length (left), number of branches (center) and area (right) of retinal vascular plexuses from tamoxifen-treated *R3*^f/f^ and *R3*^f/f^ iEC-Cre newborns. Data are represented as mean ± SEM. **C.** Immunofluorescence analysis of retinas from *R3*^f/f^ and *R3*^f/f^ iEC-Cre newborns using an endothelial (IB4, green) and a pericyte (NG2, red) marker. Representative images of twisted regions (arrows) in *R3*^f/f^ iEC-Cre veins are shown. (Lower panel) Orthogonal reconstructions of confocal Z-stack in one representative *R3*^f/f^ iEC-Cre vein showing luminal occlusion. Nuclei were stained with DAPI (blue). **D.** Representative images of arteries in retinas of *R3*^f/f^ and *R3*^f/f^ iEC-Cre pups. (Lower panel) Orthogonal reconstructions of confocal Z-stack in one representative *R3*^f/f^ iEC-Cre artery with luminal occlusion. The lumen is outlined with a white dotted line in the control. Bars are 50 μm. The p values are shown (Unpaired t-test).

In order to investigate the function of Rasa3 in ECs, we looked at postnatal retinal angiogenesis. Vascularization of the retina starts at postnatal day 0 (P0) and continues until P7-P10. *R3*^f/f^ and *R3*^f/f^ iEC-Cre newborns received tamoxifen at P1, P2 and P3 via intragastric administration and retinal vascularization was analyzed at P5. As expected, tamoxifen led to the deletion of exon 11–12 of the *Rasa3* gene (*R3*^*∆*^) specifically in *R3*^f/f^ iEC-Cre pups (Fig 2A). Body weight of control *R3*^f/f^ and mutant *R3*^f/f^ iEC-Cre pups were similar, indicating that EC deletion of Rasa3 had no major impact on postnatal growth until P5 (S2A Fig). Vascular expansion in the retina was assessed by measuring the radial progression of the vascular plexus after staining with the EC marker isolectin B4 (IB4). We observed no significant difference in the radial extension of the retinal vasculature network between *R3*^f/f^ and *R3*^f/f^ iEC-Cre pups (S2B Fig). However, vascular networks from mutant mice showed reduced complexity, compared to control mice (Fig 2B). This was confirmed by quantifying the cumulative vessel length, the number of branches and the total vascularized area of the retinal plexus, which were all significantly lower in *R3*^f/f^ iEC-Cre mice as compared with controls (Fig 2B, lower graphs).

After P3, the immature retinal vascular plexus extends and remodels into a hierarchical network, in which arteries and veins can be clearly identified. Interestingly, veins in retinas from *R3*^f/f^ iEC-Cre mice exhibited reduced diameters (S2C Fig). In addition, these vessels often displayed constricted regions and lacked a continuous lumen (Fig 2C). Arteries of *R3*^f/f^ iEC-Cre mouse retinas appeared grossly normal (Fig 2D), although their diameters were significantly increased, compared to *R3*^f/f^ mice (S2D Fig). Careful examination revealed frequent lumen occlusions (Fig 2D, orthogonal view). The lack of arterial lumen correlated with abnormal EC shape, which appeared cuboidal in *R3*^f/f^ iEC-Cre mice (Fig 2D, arrows). Interestingly, lumenization defects were also observed in a zebrafish model. Knockdown of *Rasa3* by injection of a specific morpholino in the EC specific reporter line *Tg(fli1a*:*eGFP)y1* didn’t affect the global morphology of the fish, but was associated with thinner intersegmental vessels (ISVs) and dorsal longitudinal anastomotic vessels (DLAVs) (S3A–S3C Fig). The lumen was often lacking in these vessels (S3D Fig). We also observed increased heart rate in Rasa3 morphants, which could be a compensatory mechanism for these circulatory defects (S3E Fig).

### Loss of Rasa3 affects endothelial angiogenesis and tube formation *in vitro* and *in vivo*

**Fig 3:**
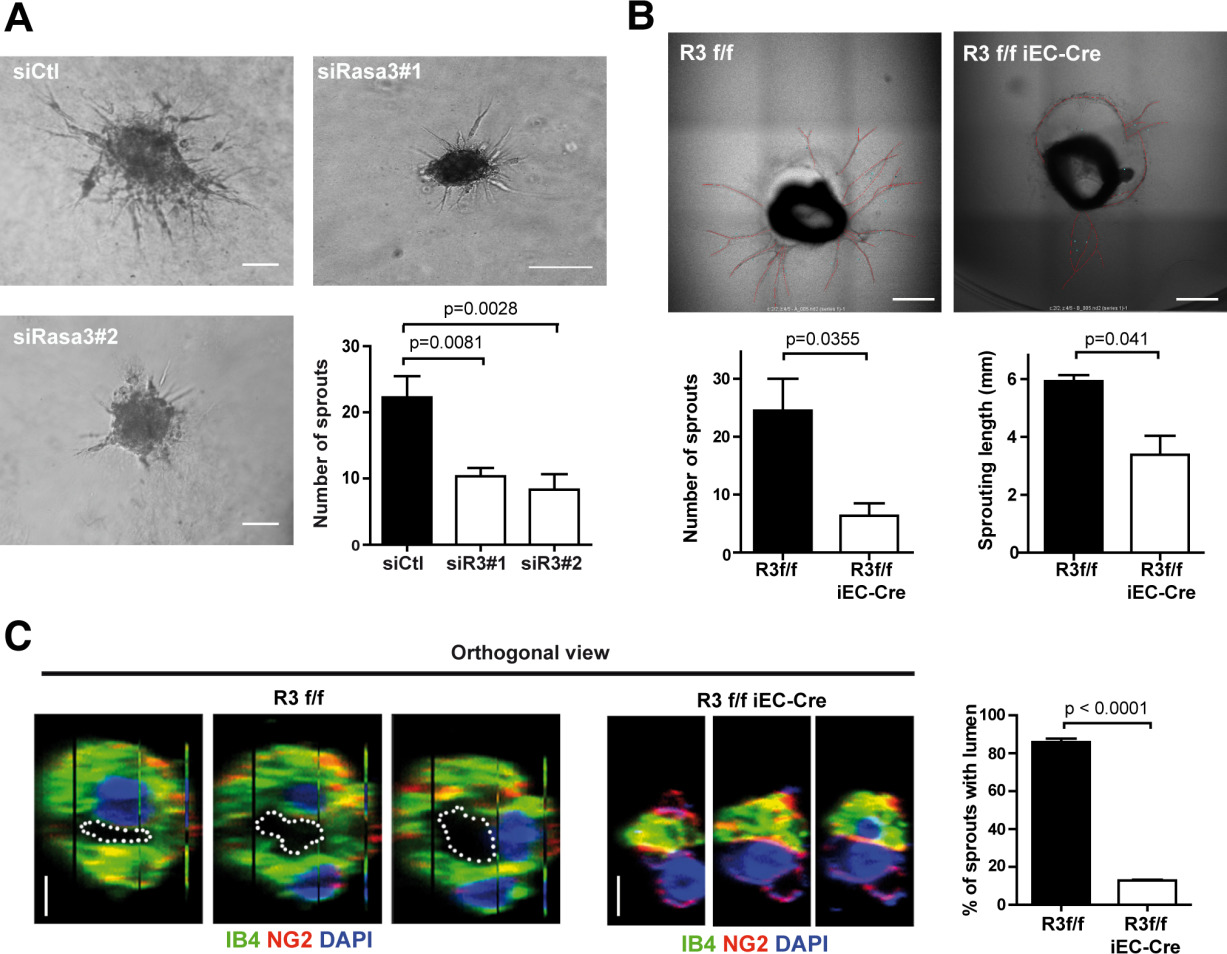
Rasa3 is necessary for endothelial sprouting and lumenization capacities. **A.** Representative micrographs of a spheroid sprouting assay with HUVECs treated with siControl or with 2 different siRasa3 (siRasa3#1 and siRasa3#2). This experiment is representative of 3 independent experiments. Bar = 100 μm. Histogram represents number of sprouts per spheroid measured on 22 and 15 spheroids, for siRasa3#1 and siRasa3#2 respectively. The p values are shown (Student’s t-test). **B.** Representative bright field images of *R3*^f/f^ and *R3*^f/f^ iEC-Cre aortic rings after 12 days of 3D collagen I-matrix culture in the presence of VEGF (10 ng/ml). New sprouts are highlighted in red. Images are representative of 5 independent experiments. (Lower graphs) Quantification of sprout number per aortic ring (left) and sprout length (right) in aortic rings isolated from *R3*^f/f^ and *R3*^f/f^ iEC-Cre mice **C.** Immunofluorescence analysis of *R3*^f/f^ and *R3*^f/f^ iEC-Cre aortic ring sprouts stained for the IB4 (green) endothelial and NG2 (red) pericyte marker. Nuclei were stained with DAPI (blue). Images are representative orthogonal reconstructions of confocal Z-stack showing collapsed lumen in *R3*^f/f^ iEC-Cre aortic ring sprout. Lumens are outlined with a white dotted line. Bars = 5 μm. (Graph) Percentage of sprouts with a lumen in *R3*^f/f^ and *R3*^f/f^ iEC-Cre aortic rings. Data are represented as mean ± SEM of 3 independent experiments. The p values are shown (Unpaired two-tail t-test).

Vascular remodeling and lumenization are dependent on physical forces exerted by blood flow [19]. To examine the effects of Rasa3 disruption outside of any potential perturbation of hemodynamic forces, we assessed the ability of human umbilical vein endothelial cells (HUVECs) deficient for Rasa3 to form capillary-like networks *in vitro*. Silencing of Rasa3 expression using two independent siRNA dramatically impaired formation of a continuous vascular-like network (S4A and S4B Fig), although it did not impact on HUVEC viability or proliferation (S4C and S4D Fig). Time-lapse microscopy showed that whereas Rasa3-deficient HUVECs initially formed branched networks, the branches were hypocellular and unstable leading to rapid collapse of the network (S1 Movie). Similar observations were made in a 3-dimensional spheroid assay, in which downregulation of Rasa3 severely impaired extension of sprouts out of the spheroid (Fig 3A). The effects of EC Rasa3 inactivation on capillary formation were also analyzed in an *ex vivo* model of adult aortic ring in which lumenized endothelial outgrowth emerging from mouse aortic explants can be examined. Inactivation of Rasa3 was achieved by daily tamoxifen ip injection of adult *R3*^f/f^ iEC-Cre mice for 5 consecutive days and isolated aortas were placed in a 3D collagen I matrix. Compared to control *R3*^f/f^ mice, the capacity of the aortic endothelium from *R3*^f/f^ iEC-Cre mice to form neovessels was dramatically impacted, with neovessels severely reduced in number and length (Fig 3B). After 12 days culture, endothelial sprouts from control *R3*^f/f^ aortas exhibited a large and well-defined lumen. In contrast, the large majority of the few sprouts that grew out of the *R3*^f/f^ iEC-Cre explants had a significantly reduced or closed lumen (Fig 3C).

### Lack of Rasa3 correlates with reduced EC adhesion turnover

**Fig 4:**
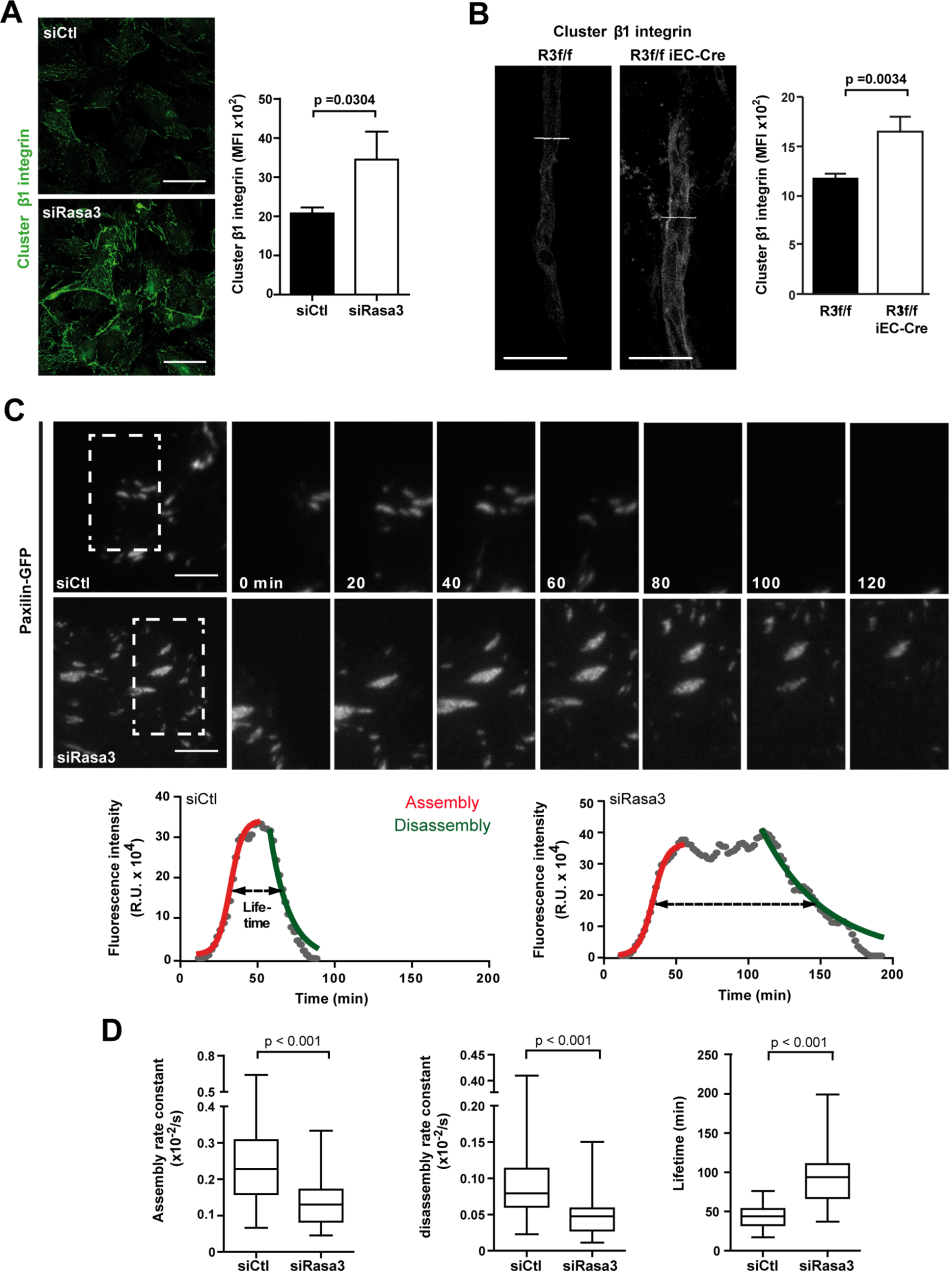
Depletion of Rasa3 impairs EC adhesion turnover. **A-B.** Activation of β1 integrin was analyzed in HUVECs transfected with control or Rasa3 siRNA **(A)** and in *R3*^f/f^ and *R3*^f/f^ iEC-Cre aortic ring sprouts **(B)** by confocal microscopy using an antibody specific for clustered β1 integrin. Representative images are shown. Bars = 50 μm. Histograms represent mean ± SD of clustered β1 integrin mean fluorescence intensity (MFI) from 3 independent experiments. The p values are shown (Student’s t-test). **C.** Time-lapse sequences of paxillin-GFP dynamics in migrating HUVECs transfected with control or Rasa3 siRNA. The regions indicated are shown at higher magnification. Graphs represent turnover dynamics in one representative adhesion experiment in control and Rasa3-depleted cells. Maximum intensity projections over the 200 min time-lapse sequences are shown as three-frame running averages. The red and green lines are respectively a logistic fit of the assembly and an exponential fit of the disassembly phase. Adhesion lifetimes are indicated by dashed arrows as defined by fluorescence intensity above the half-maximum of the fit. In the adhesion experiment shown, assembly (0,0041/s in control versus 0,0033/s in Rasa3-depleted cells) and disassembly (0,0010/s in control versus 0,0004/s in Rasa3-depleted cells) rate constants were decreased in Rasa3-depleted cells, as compared with control cells. A lag between the assembly and the disassembly was only observed in Rasa3-depleted cells. Lifetime was increased in Rasa3-depleted cells (105 min), as compared with control cells (32 min). **D.** Analysis of adhesion assembly rates, disassembly rates and lifetimes in 35 and 34 adhesions from control and Rasa3-depleted migrating HUVECs, respectively. The p values are shown (Wilcoxon—Mann Whitney test).

Although the exact molecular mechanism of vascular lumen formation and stabilization is still controversial, a common theme is the importance of EC adhesion properties. To understand how Rasa3 might control vascular lumenization, we assessed the ability of Rasa3-silenced HUVECs (siRasa3 HUVECs) to attach to major extracellular matrix (ECM) components. We found that knockdown of Rasa3 was associated with a significant increase in cell adhesion onto fibronectin (S5A Fig). In contrast, adhesion onto vitronectin, laminin or collagen was unaffected. The enhanced adhesion of siRasa3 HUVECs to fibronectin was associated with a significant increase in β1 integrin clustering (Fig 4A and S5B Fig). Interestingly, the enhanced clustering of β1 integrin was also observed in sprouts from *R3*^f/f^ iEC-Cre aortic explants (Fig 4B). Because excessive integrin clustering may reflect alterations in focal contact dynamics [20], we decided to further investigate the dynamics of adhesions in siRasa3 HUVECs. First, we examined cell migration, a process that relies heavily on assembly and disassembly of EC-ECM focal contacts (FCs). Using a scratch-wound assay, we found that downregulation of Rasa3 correlated with a significant decrease in HUVECs migratory capacity, supporting the idea that FC dynamics might be perturbed following Rasa3 silencing (S6A Fig). Cell-ECM adhesions found at membrane protrusions are usually divided into two types, depending on their maturation stage. The first adhesions to appear are nascent adhesions (NA) and focal complexes (Fx), which are small dot-like structures forming at the lamellipodium and lamellipodium-lamellum interface. While most of the Fx are unstable, a few will elongate centripetally and mature into larger (area > 1μm^2^) focal adhesions (FAs). Knockdown of Rasa3 resulted in profound alterations in the pattern of EC-ECM adhesions, as detected by labeling the FC component paxillin. Compared to control siRNA-treated HUVECs (siControl HUVECs), siRasa3 cells spreading onto fibronectin had a higher proportion of large FAs, which were localized more centripetally, whereas the number of small adhesions at the cell periphery was notably reduced (S6B and S6C Fig). Interestingly, depletion of Rasa3 also promoted accumulation of larger and more mature adhesions during VEGF-driven migration of ECs (S6D Fig).

**Fig 5:**
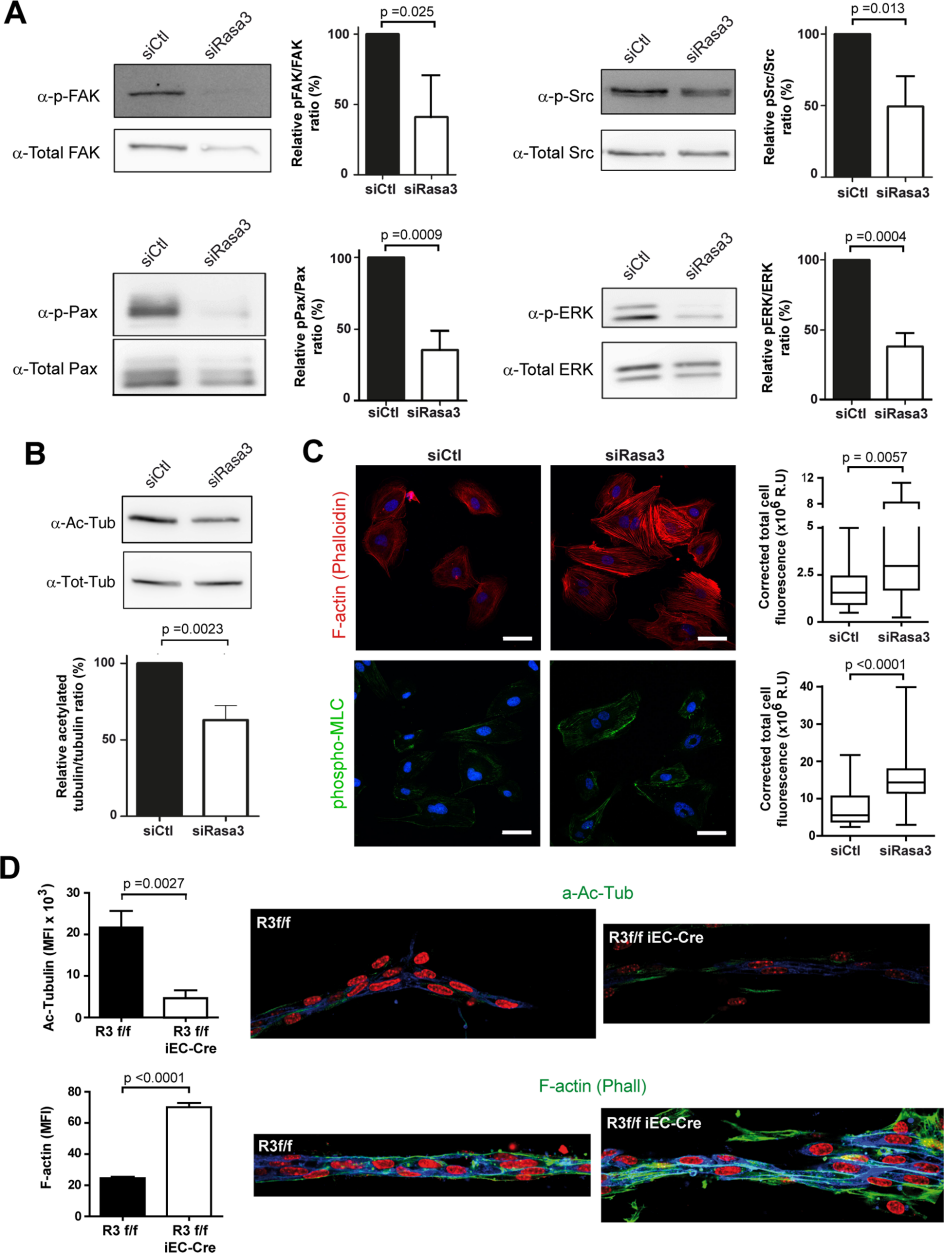
Rasa3 controls EC cytoskeleton plasticity. **A.** Detection of FAK, Src, Paxillin (Pax) and ERK phosphorylation levels in lysates from fibronectin-plated control and siRasa3-transfected cells by Western blotting. Total FAK, Src, Paxillin and ERK levels respectively were used as control. Quantifications are shown as the ratio of phospho-specific signal over total protein signal, relative to control HUVECs. **B.** Detection of tubulin acetylation levels in lysates from fibronectin-plated control and siRasa3-transfected cells by Western blotting. Total tubulin (Tot-tub) levels were used as control. Tubulin acetylation was quantified (lower) as the ratio of acetylated tubulin signal over the total tubulin signal, relative to control HUVECs. Results are expressed as mean ± SD from 3 independent experiments. **C.** Representative confocal microscopy images of control (siCtl) and Rasa3-depleted (siRasa3) HUVECs plated on fibronectin and stained for F-actin (phallodin; red) and phospho-MLC (Green). Nuclei are stained with Hoechst (blue). Bars are 50μm. Quantifications of F-actin and phospoho-MLC signals were performed on 26 cells from 3 independent experiments and are expressed as corrected mean fluorescence intensities (MFI). Results are expressed as mean ± SD from 3 independent expriments. **D.** Immunofluorescence analysis of sprouts from *R3*^f/f^ and *R3*^f/f^ iEC-Cre aortic ring stained for the IB4 (blue) and acetylated tubulin (upper) or phalloidin (lower panel) in green. Nuclei are stained with DAPI (red). Representative images of 3 independent experiments are shown. Bars = 50 μm. (Left) Quantification of acetylated tubulin (upper) and Phalloidin (lower) mean fluorescence intensity (MFI). Data are presented as mean ± SEM of 10 sprouts per group in 3 independent experiments. The p values are shown (Student’s t-test).

To analyze precisely the dynamics of adhesion assembly and disassembly during EC migration, we performed total internal reflection fluorescence (TIRF) microscopy on migrating GFP-paxillin positive HUVECs. We focused our analysis on FAs maturing just below the lamella, which appeared both larger and longer lived in siRasa3 cells (S2 Movie). We measured changes in Paxillin-GFP over time to evaluate functions for assembly and disassembly, and we determined parameters of FA dynamics, as described in Methods (Fig 4C). Assembly and disassembly rates of FA were significantly decreased in siRasa3 HUVECs (Fig 4D). As a result, FA lifetime was increased about twofold in the absence of Rasa3 (Fig 4D). Altogether, our data demonstrate that Rasa3 is important to regulate EC-ECM adhesion dynamics and stability. By initiating local tyrosine phosphorylation events, the FAK-Src signaling module is a master regulator of adhesion dynamics. This prompted us to investigate FAK/Src signaling in siRasa3 HUVECs. In agreement with decreased turnover of adhesion dynamics, we observed that reduction of Rasa3 expression was associated with diminished activation of FAK and Src following HUVECs adhesion onto fibronectin (Fig 5A) or VEGF stimulation (S7A Fig). Supporting these observations, knockdown of Rasa3 correlated with reduced phosphorylation of the downstream FAK/Src targets paxillin (Fig 5A and S7A Fig). Importantly, impaired FAK activation was also observed in EC of aortic ring sprouts from tamoxifen-treated *R3*^f/f^ iEC-Cre mice (S7B Fig).

### Rasa3 regulates EC cytoskeleton plasticity

In addition to dynamic contacts with the underlying ECM, lumen morphogenesis also requires profound plasticity of EC cytoskeleton, in order to support cell shape changes associated with expansion of the luminal compartment [21]. Tubulin acetylation, indicative of stabilized microtubules, was significantly reduced in siRasa3 HUVECs plated on fibronectin, when compared with siControl HUVECs (Fig 5B). In addition, we observed an increase in actin stress fiber level and in nonmuscle myosin IIA activity, which are known to suppress tubulin acetylation (Fig 5C). Observation of stress fibers in 3D cell culture systems such as the ring aortic assay is notoriously less conspicuous than in 2D cell culture. Nevertheless, we also observed decreased tubulin acetylation and increased stress fibers in sprouts from tamoxifen-treated *R3*^f/f^ iEC-Cre mouse aortic explants, supporting the idea that Rasa3 is important for EC cytoskeleton architecture (Fig 5D).

### Defects associated with Rasa3 depletion are mediated by Rap1 hyperactivation

**Fig 6:**
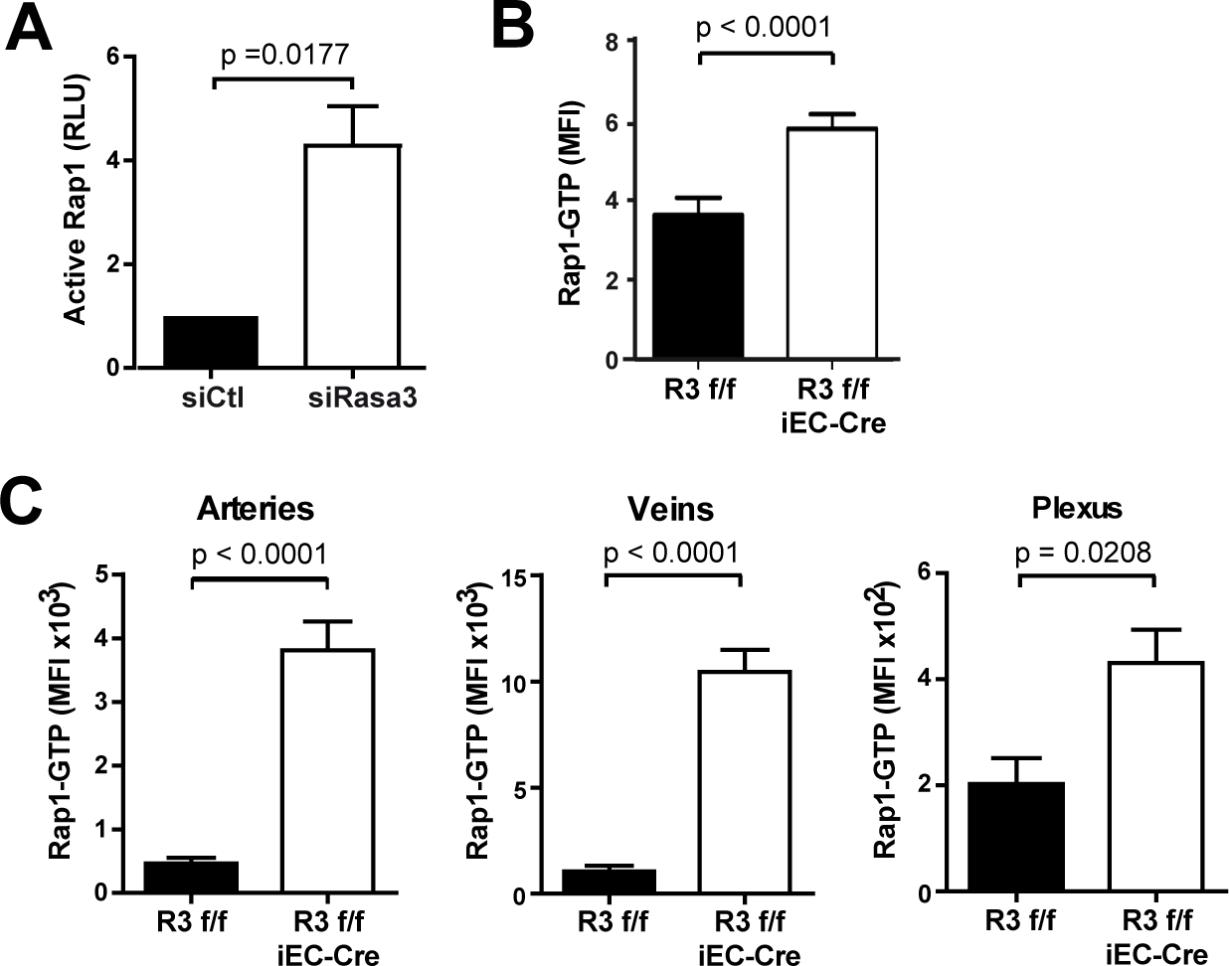
Rap1 is activated upon depletion of Rasa3. **A.** The densitometric quantification of active Rap1 detected by Western blotting on protein extracts from siControl and siRasa3 HUVECs is expressed as means ± SD from 3 independent experiments. RLU: Relative Luminescence Unit. **B.** Quantification of active Rap1-GTP mean fluorescence intensity (MFI) in aortic ring (n = 10) sprouts from *R3*^f/f^ and *R3*^f/f^ iEC-Cre mice (n = 10). Data are epresented as mean ± SEM. **C.** Quantification of active Rap1-GTP mean fluorescence intensity (MFI) in arteries, veins and plexus of *R3*^f/f^ and *R3*^f/f^ iEC-Cre newborn retinas (n≥8). Data are represented as mean ± SEM. The p values are shown (Student’s t-test).

Based on our recent finding that in megakaryocytes Rasa3 controls Rap1 but not R-Ras activation [17], we assessed the levels of these active small GTPases in ECs lacking Rasa3. Decreasing Rasa3 expression in HUVECs significantly increased active Rap1 levels (Fig 6A), but had no effect on R-Ras levels (S8 Fig), as reported in megakaryocytes. Analysis of aortic sprouts from *R3*^f/f^ and *R3*^f/f^ iEC-Cre aortic explants also showed that deletion of Rasa3 correlated with significantly higher levels of active GTP-bound Rap1 (Fig 6B). These observations were also confirmed *in vivo*, as active Rap1 levels were dramatically higher in arteries, veins, and plexus of *R3*^f/f^ iEC-Cre retina, compared to *R3*^f/f^ retina (Fig 6C).

Rap1 is involved in the activation of β1-integrins in ECs and plays a key role in integrin-dependent angiogenic functions of ECs such as sprouting, migration and adhesion [14], all of which are affected by Rasa3 depletion in ECs. In addition, Rap1 is known to promote stability of endothelial VE-cadherin-based cell- cell junctions [22]. ECs display two types of VE-cadherin containing junctions [23]. Junctions of the first type localize linearly along cell-cell borders and are considered as stable adherens junctions (AJs). Junctions of the second type appear as short linear structures that are almost orthogonal to the cell-cell borders and are remodeling junctions called focal AJs (FAJs). Quantification of the total length of FAJs in single cells relative to the total junction length revealed that siRASA3 HUVECs had a reduced proportion of FAJs, indicating that cell-cell junctions are more stable when Rasa3 is knockdown, consistent with increased Rap1 activity (S9A Fig). In line with this, we found that junctions of Rasa3 knockdown HUVECs were more resistant to the cell-cell junction-destabilizing agent EGTA than those from control cells. Whereas VE-cadherin was completely internalized in control cells after 5 min of EGTA treatment, it still partially localized at the cell membrane in siRasa3 HUVECs, indicative of more resilient cell-cell junctions (S9B Fig). In addition, EGTA-induced vascular permeability was also reduced in Rasa3-deficient HUVECs, as measured by assessing solute flux across an EC monolayer (S9C Fig). Through phosphorylation of VE-cadherin, Src has emerged as a prominent mediator of VE-cadherin-mediated AJ destabilization and vascular permeability [24]. Consistent with their more stable cell-cell junctions, siRasa3 HUVECs showed reduced phosphorylation of VE-cadherin Y658 and Src activation (S9D Fig).

**Fig 7:**
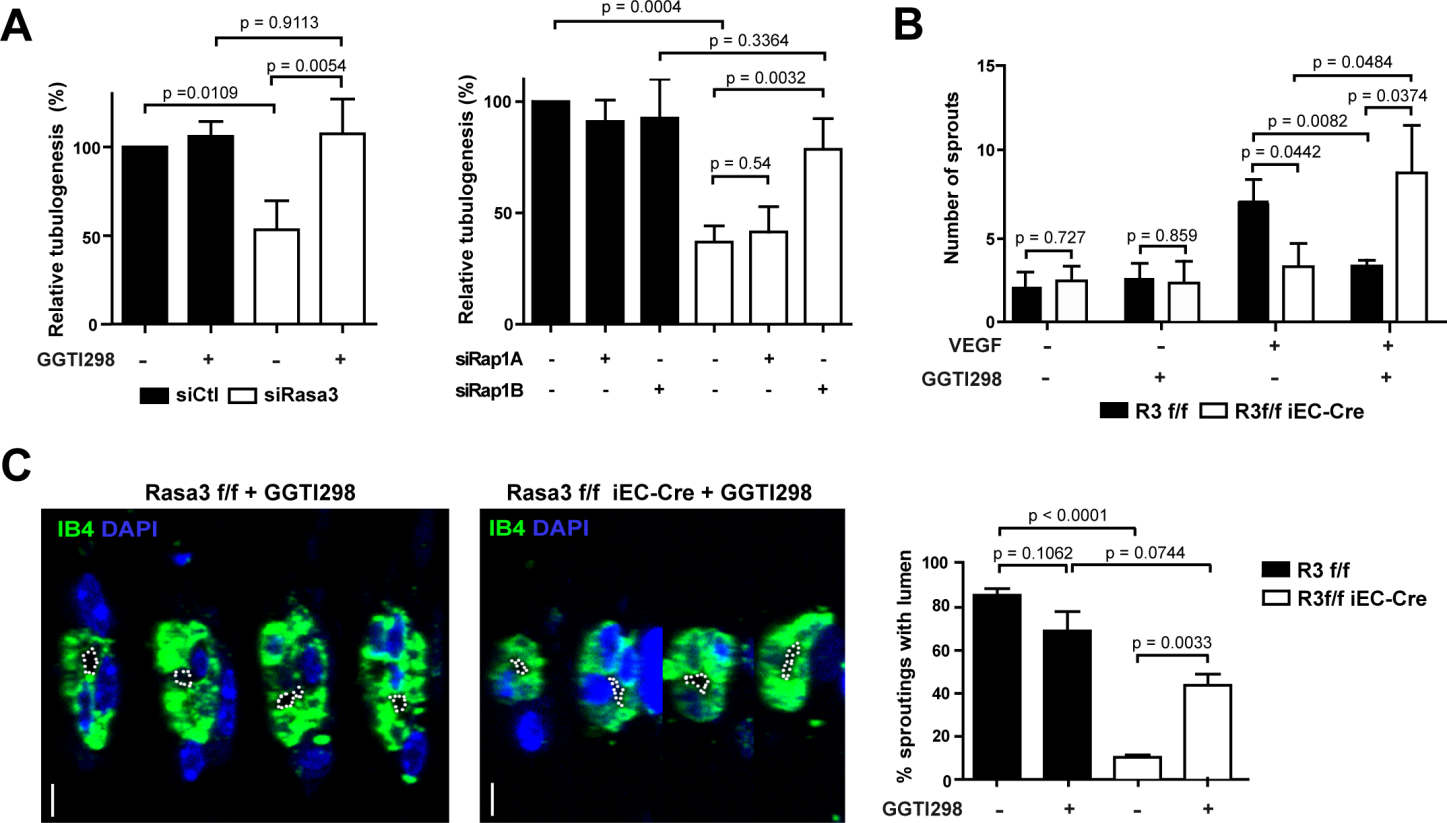
Inactivation of Rap1 rescues the Rasa3-depleted phenotypes. **A.** Quantification of capillary-like network formation in siControl vs siRasa3 HUVEC treated or not with GGTI298 (left) or co-transfected with siRap1A, siRap1B or a second control siRNA (right). Results are expressed as mean ± SD cumulative length of capillary-like structures measured in 5 different fields per experiment from 3 independent experiments, relative to non-treated siControl HUVECs (left) or to double siControl HUVECs (right), respectively. **B.** Number of sprouts growth out from *R3*^f/f^ and *R3*^f/f^ iEC-Cre aortic rings after 8 days of culture in the presence or absence of the Rap1 inhibitor GGTI298. A minimum of 10 aortic rings were analyzed per group in 3 independent experiments. **C.** Representative orthogonal reconstruction images of confocal Z-stacks from *R3*^f/f^ and *R3*^f/f^ iEC-Cre aortic ring sprouts, in the presence or absence of the Rap1 inhibitor GGTI298, stained for the IB4 (green) endothelial and nuclei DAPI (blue). Lumens are outlined with a white dotted line. Bars = 5 μm. Graph represent the mean ± SEM of the percentage of lumenized sprouts from *R3*^f/f^ and *R3*^f/f^ iEC-Cre aortic rings cultured in the same conditions as in panel B. The p values are shown (Student’s t-test).

In order to test whether suppression of Rap1 by Rasa3 played a role during EC lumen formation, we inhibited Rap1 activity in siRasa3 HUVECs. Tubulogenesis defects of siRasa3 HUVECs were completely reverted upon treatment with the Rap1 inhibitor GGTI298 (Fig 7A, left graph). These results were further confirmed when Rap1 hyperactivation was prevented with suboptimal concentrations of either Rap1a or Rap1b siRNAs. Neither siRap1a nor siRap1b alone had an effect on *in vitro* tubulogenesis of control HUVECs. However, siRap1b, but not siRap1a, almost completely rescued the tubulogenesis defects in Rasa3 deficient HUVECs (Fig 7A right graph). Rap1 inhibition by treatment with GGTI298 increased by almost threefold the number of sprouts from *R3*^f/f^ iEC-Cre aortic explants, while it dramatically reduced the sprouting ability of control *R3*^f/f^ aortic rings (Fig 7B). More importantly, inhibition of Rap1 also increased the number of sprouts from *R3*^f/f^ iEC-Cre aortic rings that show a visible lumen (Fig 7C). Moreover, in the zebrafish model, the lumen defects were also partially rescued in presence of the GGTI298 Rap1 inhibitor (S3F Fig). Altogether, these observations demonstrate that Rasa3 controls endothelial lumenization by regulating Rap1 dependent signaling.

## Discussion

Here, using a combination of *in vitro* cell biology approaches and loss of function studies in mouse and zebrafish, we identify a key role for Rasa3 in the maintenance of vascular integrity in vertebrates. We show that deletion or knockdown of Rasa3 in ECs is associated with hyperactivation of the small GTPase Rap1 and deregulation of EC adhesion properties. When Rasa3 is deleted or knocked down, turnover of β1 integrin-dependent EC adhesion is impaired and EC-ECM basal adhesion contacts accumulate. In addition, EC-EC adhesions are stabilized, leading to decreased endothelial permeability. These adhesion defects prevent formation of a patent lumen and result in occluded blood vessels, hemorrhages and early embryonic death in EC-restricted *Rasa3* KO mice.

We and others have shown that mice expressing inactive mutants of Rasa3 die during mid-embryonic life and display hemorrhages and severe thrombocytopenia resulting from developmental defects during megakaryopoiesis [16–18]. Because low levels of platelets could potentially explain embryonic bleeding and mortality, we generated mice specifically inactivated for *Rasa3* in the megakaryocyte lineage (*R3*^f/f^ PF4-Cre). As expected, these mice display megakaryocyte alterations and a severe thrombocytopenia. Surprisingly, *R3*^f/f^ PF4-Cre newborn mice were obtained at Mendelian ratios and were viable, although with reduced life span (S1 Fig and S1 Table). A recent study from Stefanini et al. independently reported similar observations. However, these authors observed that the hemorrhagic phenotype in *R3*^f/f^ PF4-Cre embryos was much less severe than in *R3*^*-/-*^ embryos, suggesting that embryonic bleeding and lethality associated with *Rasa3* inactivation might relate to its function in a cell compartment different from the megakaryocyte lineage. Here, we show that EC-specific deletion of *Rasa3* results in the same lethal phenotype as in full *R3*^*-/-*^ embryos, indicating that the specific requirement for Rasa3 during mouse embryonic development is largely linked to its function in the developing vascular endothelium. EC Rasa3 is thus essential to maintain normal blood vessel tubulogenesis and vascular integrity *in vivo*. Numerous studies have documented that vascular lumen instability or occlusions often lead to hemorrhages and mid- or late gestation embryonic lethality [3,25–27].

Rasa3, like every member of the GAP1 GTPase family besides Rasa2, has the ability to control both R-Ras and Rap1 small GTPases *in vitro* [13]. *In vivo*, the specificity of Rasa3 towards R-Ras or Rap1 remains unclear. *Scat* mice, bearing the G125V mutation in Rasa3 show increased R-Ras activity in erythrocytes, which could explain the delayed erythropoiesis phenotype [15]. In megakaryocytes and platelets, we and others have shown that Rasa3 deletion leads to upregulation of Rap1 activity without affecting R-Ras activity [17,18]. Here, we show that the absence of Rasa3 in ECs correlates with increased Rap1 activity *in vivo* and in cultured endothelial cells. By contrast, no effect was observed on active R-Ras levels when Rasa3 was knockdown in the later cells. Importantly, inhibition of Rap1 using the GGTI298 inhibitor or specific siRNA rescued adhesion and tubulogenesis defects. These observations thus identify Rap1, and not R-Ras, as the main target of Rasa3 in ECs and are consistent with the idea that Rap1 and R-Ras largely act in different signaling pathways and are selectively regulated by specific GAPs and GEFs *in vivo* [28].

Rap1 signaling has been associated with multiple aspects of vascular development and endothelial cell biology [14]. As for other cell types, Rap1 is predominantly involved in the control of integrin and cadherin-mediated adhesion dynamics in endothelial cells [29,30]. In mice, EC specific inactivation of Rap1 leads to hemorrhage and vascular rupture. More interestingly, these mice exhibit microvessel dilation [31]. In cultured endothelial cells, depletion of Rap1 diminishes adhesion to the ECM, promotes VE-cadherin-based cell-cell junction remodeling and increases endothelial permeability [32,33]. These effects have been partly linked to the role of Rap1 in the regulation of integrin β1 affinity and clustering [14]. All these described Rap1 functions are entirely consistent with the phenotype of Rasa3-depleted HUVECs, which exhibit Rap1 hyperactivation and concomitantly increase in β1 integrin clustering and decrease in focal adhesion dynamics, permeability and cell-cell junction remodeling. Remarkably, decreasing expression of Rap1 annihilates EC tubulogenesis *in vitro*, similarly to depleting Rasa3 [33]. This supports the idea that tube formation relies on a tight balance of EC adhesion dynamics and identifies Rasa3-Rap1 signaling as a critical hub in this process.

A recurrent theme in endothelial tubulogenesis is the coordinated control of adhesion processes and cytoskeleton dynamics of ECs [34]. In the model of cord hollowing, the initial VE-cadherin-based AJs between ECs relocalize laterally to allow initial opening of the lumen [26]. It is likely that accumulation of VE-cadherin-based EC-EC junctions towards the cord periphery is achieved through VE-cadherin internalization at the apical cell surface and recycling at the lateral positions, requiring coordinated VE-cadherin phosphorylation events [35]. In this regard, our observation that Rasa3-depleted HUVECs exhibit stable VE-cadherin-based AJs and decreased phosphorylation of VE-cadherin Y658 is consistent with their lower FAK/Src signaling, as both kinases have been extensively documented to increase VE-cadherin phosphorylation and promote EC junction turnover. VE-cadherin also influences actin cytoskeleton remodeling, which is required for the EC shape changes necessary to accommodate the growth of the luminal compartment. VE-cadherin signaling thus plays a critical role in vascular tubulogenesis, as illustrated by the lumenization defects observed in VE-cadherin-deficient mice and zebrafish [36,37].

Lumen expansion also requires ECs to establish dynamic contacts with the underlying ECM. Loss of β1 integrin during development of the mouse vascular network prevents lumen formation in medium and small sized arteries [3]. In contrast, we have previously shown that excessive stability of EC-ECM adhesions impairs ISV lumenization in zebrafish [4]. Together with our observations here, it is thus becoming evident that a tight regulation of adhesion complexes between ECs and the ECM is required to allow vascular lumen formation and maintenance. Recent observations support the existence of crosstalk between integrin-based cell-matrix and cadherin-based cell-cell contacts, both of which may operate separately on lumen formation or stabilization [3,38,39]. Strikingly, a number of these studies have converged on Rap1, thus placing this small GTPase at the crossroads of multiple outside-in and inside-out adhesion signaling pathways [40].

**Fig 8:**
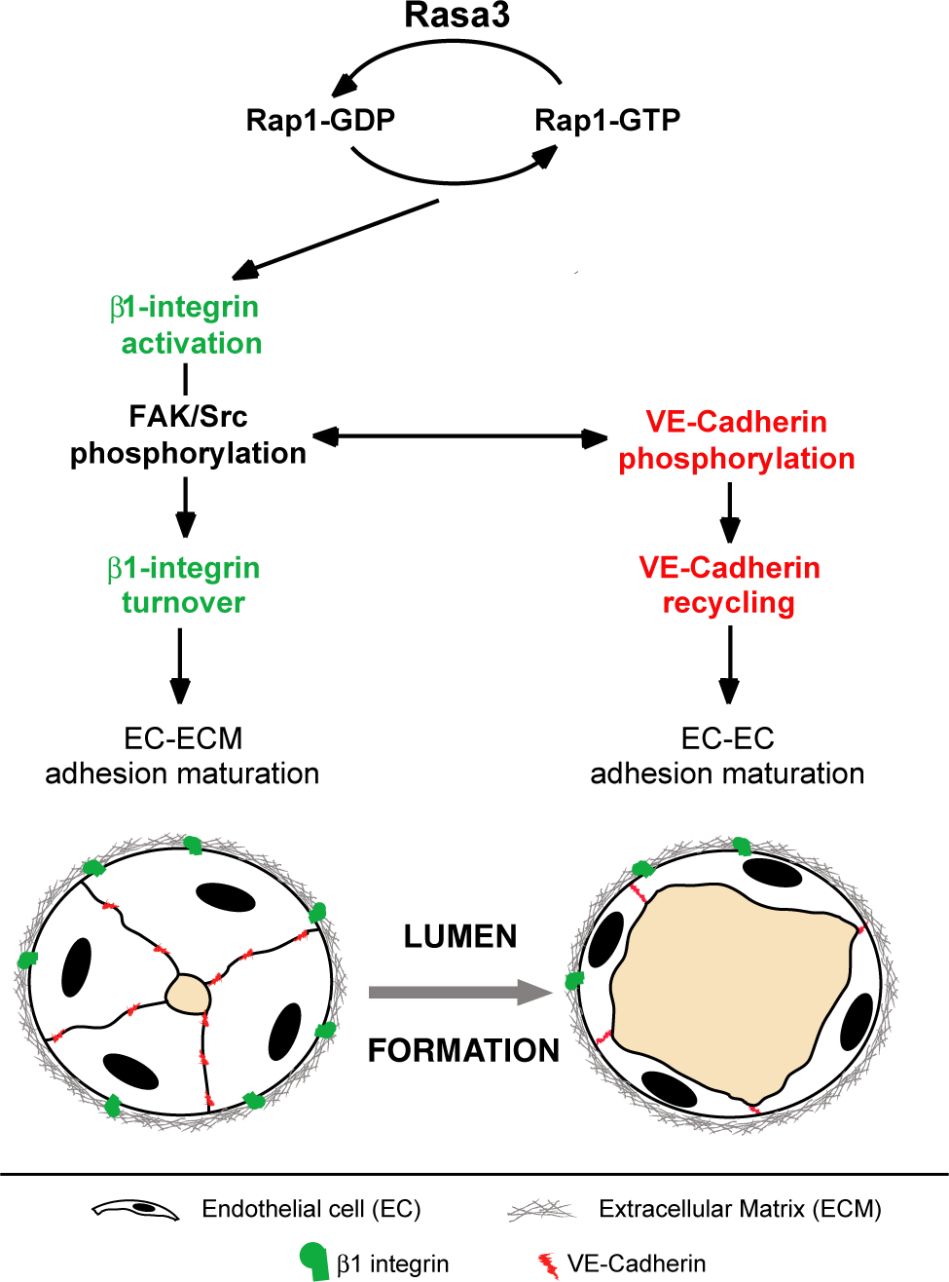
Model for Rasa3 control on endothelial lumen formation. Rap1 inactivation by Rasa3 GAP activity regulates activation of β1 integrin- and VE-cadherin-based adhesions. Following integrin activation, Rasa3 inactivates Rap1 to allow turnover of integrin- and VE-cadherin-based adhesions, via the FAK/Src signalling module. Failure to turnover and recycle EC-ECM and junctional adhesion complexes between EC results in vascular tubulogenesis defects. Thin black arrows indicate signalling pathways. The two-way thin black arrow indicates the interaction between the FAK/Src signalling module and the VE-cadherin signalling pathway.

The developmental consequences of EC Rasa3 ablation in mice, as dissected in this study, shed light on the critical function of this poorly characterized protein in vertebrate development. The first and main cause of embryonic lethality associated with Rasa3 inactivation appears to be excessive activation of Rap1, which leads to dysregulation of EC adhesion properties and signaling. As a result, Rasa3-depleted ECs are unable to integrate and coordinate integrin and VE-cadherin signaling, preventing formation of a functional lumen. Our results thus uncovered an important but previously unknown coordinator of multiple adhesion processes during vascular tubulogenesis (Fig 8). Better understanding of the intricate molecular networks linked to vascular lumen formation should pave the way towards new vascular-targeted therapies.

## Methods

### Ethics statement

Studies on mice were conducted according to internationally-accepted standards. Mice studies were authorized by the Animal Care Use and Review Committee of the University of Liege (approval numbers: 1517, 1628 and 1902).

### Mice

*Rasa3*^flox/+^ (*R3*^f/+^) mice with exons 11 and 12 of the Rasa3 gene flanked by LoxP sites were generated by inGenious Targeting Laboratory, Inc. *R3*^f/+^ mice were crossed with PGK-Cre mice to generate *R3*^∆/+^ mice as well as with PF4-Cre mice for megakaryocytes and platelets deletion and with Cdh5(PAC)-CreERT2 (or iEC-Cre) mice for deletion in EC in the presence of tamoxifen [41]. All mice were housed in an animal facility with a 12 h light/12 h dark cycle and had free access to food and water throughout the study period.

### Tamoxifen treatments

For studies on embryos, plugged female *R3*^f/f^ mice were ip injected at E8.5 and E10.5 with tamoxifen. For retinal angiogenesis studies in *R3*^f/f^ and *R3*^f/f^ iEC-Cre newborns, intragastric administration of tamoxifen was performed at P1, P2 and P3, according to Pitulescu et al [42]. For aortic ring studies in adult mice, 10 week-old *R3*^f/f^ and *R3*^f/f^ iEC-Cre mice were ip injected with tamoxifen for 5 consecutive days. After tamoxifen treatment, DNA recombination was assessed by PCR detection of the *R3*^∆^ allele on tail DNA.

### Antibodies and reagents

A list of the primary and secondary antibodies used in this study is presented in S2 Table. TRITC-phalloidin served for F-actin staining (Molecular Probes). DAPI and FITC-Dextran (FD405) were obtained from Sigma. Detection of active GTP-bound Ras and Rap1 in HUVECs was performed with Cell Signaling kits (#8821 and #8818, respectively). The low molecular Rap1 inhibitor GGTI298 was from Sigma. Control (SR-CL000-005) and Rasa3 (5’-GCGCTTTGGGATGAAGAAT-3’ and 5’CCTGAAGTTTGGAGATGAA-3’) siRNAs were purchased from Eurogentec. Tamoxifen was from Sigma, VEGF was from Peprotech, fibronectin and collagen I were from Gibco, vitronectin was from Millipore and laminin was from Invitrogen.

### Whole mount newborn retina assay

For whole mount retina immunofluorescence, we proceeded as Pitulescu et al [42]. Eyes were fixed in 4% paraformaldehyde in PBS at 4°C overnight and washed in PBS. Retinas were dissected, permeabilized in PBS, 1% BSA, 0.5% Triton X-100 at 4°C overnight, rinsed in PBS, washed twice in PBlec (PBS, pH 6.8, 1% Triton-X100, 0.1 mM CaCl2, 0.1 mM MgCl2, 0.1 mM MnCl2), and incubated in biotinylated isolectin B4 (Sigma- Aldrich), 20 μg/ml in PBlec at 4°C overnight. After five washes in PBS, samples were incubated with streptavidin conjugates (Alexa-488, -594, or -647; Molecular Probes) diluted 1:400 in PBS, 0.5% BSA, 0.25% Triton X-100 at room temperature for 3 h. After washing and a brief postfixation in paraformaldehyde, the retinas were either flat-mounted using Prolong (Molecular 2 probes) or processed for multiple labeling. DAPI (1:5000, 20 mg/ml, Sigma) was used for nuclear staining. Flat-mounted retinas were analyzed by confocal laser scanning microscopy using a Nikon A1 microscope. Images were processed using ImageJ. Fields of views at the sprouting vascular front of the retinal vascular network, including regions of capillary-sized vessels directly adjacent to radial arterioles, were captured using a 40x objective lens. For quantification, at least four fluorescent images/retinas were taken from 8 mice per group.

### Aortic ring assay

For aortic ring *ex vivo* culture assay, we proceeded as Baker et al [43]. Freshly dissected aortas isolated from 12 week-old mice were placed in ice-cold OptiMEM, cleaned of fatty tissue under a dissecting microscope, and rinsed in ice-cold OptiMEM three times to remove residual blood before they were sliced into 0.5-mm-thick rings, using a surgical scalpel. The rings were starved overnight at 37°C in a 5% CO2 humidified incubator before they were embedded in 50 μl of Collagen I matrix. Matrix was overlaid with 100 μl of OptiMEM supplemented with Glutamine, 10% FBS and antibiotics. New sprouts from the rings were induced by adding VEGF (10 ng/ml) to the culture medium for 12 days, with medium change every 2 days. The outgrowth and branching activity of endothelial tubes were counted using a Nikon inverted microscope. A minimum of twelve rings per genotype were used for each assay and each assay was repeated at least 4 times. Aortic rings were immunostained according to Baker et al. 5. Briefly, aortic rings were fixed in 4% paraformaldehyde in PBS and washed in PBS. After, they were permeabilized in PBS, 1% BSA, 0.5% Triton X-100 at 4°C overnight, rinsed in PBS, washed twice in PBlec (PBS, pH 6.8, 1% Triton-X100, 0.1 mM CaCl2, 0.1 mM MgCl2, 0.1 mM MnCl2), and incubated in biotinylated isolectin B4 (Sigma- Aldrich), 20 μg/ml in PBlec at 4°C overnight. After five washes in PBS, samples were incubated with streptavidin conjugates (Alexa-488, -594, or -647; Molecular Probes) diluted 1:400 in PBS, 0.5% BSA and 0.25% Triton X-100 at room temperature for 3 h. After washing and a brief post-fixation in paraformaldehyde, the aortic rings were either flat-mounted using Prolong (Molecular probes) or processed for multiple labeling. DAPI (Sigma) served for nuclear staining. Flat mounted aortic rings were analyzed by confocal laser scanning microscopy using a Nikon A1 microscope.

### Blood platelets quantification

Eight to twelve week-old male mice were bled under sodium pentobarbital anesthesia from the retro-orbital plexus in EDTA containing tubes. Blood platelets were quantified with a Cell Dyn 3500 analyzer (Abott Diagnostic).

### Zebrafish

Knockdown experiments were performed by injecting embryos at the one-cell stage with 2.5 ng of Control (Ctl) or Rasa3 morpholino. Ctl (5'-CCTCTTACCTCAGTTACAATTTATA-3') and Rasa3 (5’-AAGCCCTTCTTCTTCGACCGCCATG-3’) morpholinos were purchased from Genetools. The injected embryos were placed in E3 medium and incubated at 28°C. Confocal pictures were taken on living embryos using a Zeiss confocal microscope at 48 hpf (hours post fertilization). The Tg(fli1a:eGFP)y1 fish were maintained conforming to EU regulations on laboratory animals. For the rescue experiment, the GGTI298 (10 μM) was added to the E3 medium at 40 hpf until 48 hpf when the quantification was made.

### HUVEC culture and siRNA transfection

Human Umbilical Vein Endothelial Cells (HUVECs) were obtained from Lonza and grown at 37°C, in 5% CO_2_. SiRNA transfections in HUVECs were performed using the GeneTrans 2 (MoBiTec) reagents according to the manufacturer's protocol.

### Tube formation assay

48 hours after transfection, HUVECs were subjected to the Matrigel assay as described Martin et al, 2008 [44]. 50 x 10^3^ cells were cultivated for 16 h on 220 μl of Matrigel Basement Membrane Matrix (BD Bioscience). Quantification was done by measuring the cumulative tube length in three random microscopic fields using ImageJ software.

### Wound healing

72 h after transfection, a confluent monolayer of HUVECs was scraped in order to create a cell free zone. Quantification of cell migration was done by measuring the percentage of recolonized area 8h after injury using the ImageJ software.

### Adhesion assay

72 h after transfection, HUVECs were incubated in fibronectin-, collagen 1-, vitronectin-, or laminin-precoated coverslips for 30 min. The cells were washed and stained with crystal violet. Relative adhesion is measured by reading absorbance at 560 nm after release of incorporated dye.

### Focal adhesion dynamics

Focal adhesion dynamics and quantifications were performed as described by Stehbens and Wittman [45]. HUVECs were co-transfected with siRNA and paxillin-GFP. Focal adhesion dynamics were analyzed using time-laps TIRF (total internal reflection fluorescence) microscopy. Live-cell TIRF imaging was performed on a Nikon Eclipse Ti-E inverted microscope equipped with perfect focus system, CFI Apo TIRF 100X oil objective (Nikon), a QuantEM 512SC EMCCD camera (photometrics, Roper Scientific), a TI-TIRF-E motorized TIRF illuminator (Nikon) and a stage top incubator maintaining 37°C and 5% CO2 (Tokai hit). Images were captured every 120 seconds over 10 h.

### Permeability assay

One day after transfection, 6 x 10^4^ HUVECs were plated onto insert (FisherBioblock W2127C) precoated with fibronectin. The insert defined a top and a bottom chamber. After transfection (72h), the medium was changed in both chambers, and cells were treated with a medium containing EGTA (4mM) and FITC-Dextran (1mg/ml) for 30 min. Samples from the bottom chamber were analyzed by measuring the fluorescence at 492nm.

### Confocal imaging analysis

For lumen quantification, orthogonal reconstructions of the Z-stacks were generated. ImageJ was used for quantification of fluorescence intensity, distances and surfaces. In adhesion experiments, HUVECs were seeded for 30 min onto fibronectin-coated coverslips, 72h after siRNA transfection. In VEGF treated experiments, HUVECs were seeded 48 h after siRNA transfection and, the next day, were treated during 5 min with VEGF (50 ng/ml) and processed for imaging. For EGTA-treated experiments, HUVECs were seeded 24 h after siRNA transfection in order to have a confluent monolayer of cells. Two days after, cells are treated for 5 min with EGTA (4 mM) and processed for imaging. For confocal analysis, cells were fixed in 4% paraformaldehyde or 100% methanol, permeabilized in 0.1% Triton X-100, blocked in BSA and incubated overnight with the appropriate primary antibodies. Samples were then incubated with the corresponding Alexa-conjugated secondary antibodies (Invitrogen) and, after washing, mounted with Mowiol. All images were acquired with a Nikon A1 confocal microscope. The average size and the adhesion size distribution of focal adhesions were measured using the “analyze particles” plugin of ImageJ software. For *in vivo* and *ex vivo* experiments, Z-sections were acquired for precise structure and fluorescence intensity analyses with ImageJ. All images in the same experiment were acquired and analyzed in the same conditions.

### SDS-PAGE and western blotting

Cells were harvested and total extracts were obtained by lysing cells in Laemmli buffer containing 50mM of Dithiothreitol (DDT). SDS-PAGE and Western Blot analysis were performed according to standard procedures and developed with ECL detection kit (GE Healthcare Bio-Sciences). Quantification of bands intensity was determined with ImageJ software.

### FACS analysis

72 hours after transfection, cells were washed three times in blocking solution (PBS 1% FBS) and incubated for one hour with the anti-activated β1 integrin primary antibody. Cells were then washed with blocking solution and incubated with a FITC-conjugated secondary antibody. After washing, the expression of the clustered β1 integrin was quantified by flow cytometry using FACScan Cytometer (Becton Dickinson).

**Statistics.** Statistical analyses were performed with Graphpad Prism 3.0. The test used for each experiment is described in the corresponding legend. For each test, a difference of P<0.05 was considered significant.

## Supporting information

S1 Fig Severe thrombocytopenia but no embryonic lethality in *R3*^f/f^ PF4-Cre mice in which *Rasa3* is specifically inactivated in megakaryocytes and platelets. **A.** Immunodetection of Rasa3 and γ-Tubulin by Western blotting on washed-platelet extracts isolated from blood of *R3*^f/f^ (1) and *R3*^f/f^ PF4-Cre (2). Image is representative of 5 independent experiments. **B.** Survival curve of *R3*^f/f^ (n = 11) and *R3*^f/f^ PF4Cre (n = 8) mice over a period of 50 weeks. The p value is shown (Log-rank (Mantel-Cox) test). **C.** Blood platelet counts in adult *R3*^f/f^ (n = 8) and *R3*^f/f^ PF4-cre (n = 8) mice. Data are represented as mean ± SEM. The p value is shown (Unpaired t-test). **D.** Quantification of megakaryocytes present in the spleen of *R3*^f/f^ (n = 6) and *R3*^f/f^ PF4-Cre (n = 6) mice. Data are represented as mean ± SEM; the p value is shown (Unpaired t-test).
(TIF)

S2 Fig Deletion of Rasa3 in *R3*^f/f^ iEC-Cre newborns by daily tamoxifen injections results in vascular defects. **A.** Quantification of *R3*^f/f^ (n = 18) and *R3*^f/f^ iEC-Cre (n = 8) newborn body weight at P5 after tamoxifen treatment. **B.** Representative image of *R3*^f/f^ (n = 18) and *R3*^f/f^ iEC-Cre (n = 8) newborn retina vasculature, stained with the IB4 (green) endothelial marker. The blue line and the white arrow indicate the total length and the vascular front of the vascular network, respectively. Bars = 2 mm. Quantification of the radial outgrowth of the retinal network is shown. **C**-**D.** Quantification of the vein (**C**) and arteries (**D**) diameter in P5 newborn retinas of *R3*^f/f^ (n = 8) and *R3*^*f*/f^ iEC-Cre (n = 8) newborns. Data are represented as mean ± SEM. The p values are shown (Unpaired t-test).
(TIF)

S3 Fig Knockdown of Rasa3 in Zebrafish induces tubulogenesis defects in the trunk vasculature. **A.** Detection of Rasa3 level in lysates from Control (Ctl) and Rasa morphant embryos. GAPDH was used as control. **B.** General morphology of Ctl and Rasa3 morphant embryos. **C.** Tg(fli1a:eGFP)y1 embryos were injected with control morpholino (MoCtl) or with morpholino targeting Rasa3 (MoRasa3). Confocal pictures of the trunk vasculature were taken at 48 hpf. Ctl embryos present normal ISVs and DLAVs with open lumen (arrow head) whereas Rasa3 morphant embryos show thinner, non-lumenized vessels (arrows). Bars = 50 mm. ISV, intersegmental vessel; DLAV, dorsal longitudinal anastomotic vessels. **D.** Quantification of lumenized ISVs in Ctl and Rasa3 morphant embryos at 48 and 72 hpf. The p values are shown (Fisher's exact test). Results are mean from 10 ISVs/embryo in 50 embryos at 48hpf and 25 embryos at 72hpf. **E.** Heart rates in 32 Ctl and 30 Rasa3 morphant embryos. Histograms are mean ± SD from 35 embryos. The p value is shown (Fisher's exact test). **F.** Rescue experiment using GGTI298 (10 μM). Quantification of lumenized ISVs in Ctl and Rasa3 morphant embryos at 48hpf. The p values are shown. Results are mean from 10 ISVs/embryo in 35 embryos.
(TIF)

S4 Fig Deletion of Rasa3 impairs tubulogenesis in HUVECs. **A.** Immunodetection of Rasa3 and Actin by Western blotting on total extracts from HUVECs transfected with a control siRNA or two different Rasa3 siRNAs (siRasa3#1 and siRasa3#2). **B.** Representative micrographs of a tube-like formation assay in Matrigel using HUVECs treated with siControl or with two different Rasa-siRNA (siRasa3#1 and siRasa3#2). Images are representative of 3 independent experiments. Bar = 100 μm. Histograms represent mean ± SD of relative tubulogenesis of capillary-like structures measured in five different fields from 3 independent experiments. The p values are shown (One sample t-test). **C.** Histograms represent mean relative cell viability ± SD in siCTL or siRasa3-treated HUVECs from 3 independent experiments. **D.** Histograms represent mean cell number of siRasa3#1 at indicated time points after seeding and relative to the number of cells in siCTL-treated HUVECs. Results are from at least 3 independent experiments. The p values are shown in **B**, **C** and **D** (Student’s t-test).
(TIF)

S5 Fig Depletion of Rasa3 increases adhesion and decreases migration of ECs. **A.** Effects of Rasa3-silencing on HUVEC adhesion onto Fibronectin, Collagen, Vitronectin or Laminin. Bars = 100 μm. Representative micrograph of an adhesion assay with HUVECs treated with siCTL or siRasa3. Images are representative from 3 to 5 independent experiments. Histograms are mean ± SD of 3 independent experiments. The p values are shown (Student’s t-test). **B.** Immunodetection of total integrin β1 levels by Western blotting on total extracts from HUVECs transfected with control or Rasa3 siRNA (related to the experiment described in Fig 5A). Actin was used as a loading control.
(TIF)

S6 Fig Rasa3 is required for normal adhesion turnover. **A.** In a scratch-wound migration assay, the recolonized area was analyzed at 5h in HUVECs transfected with siControl or two different siRasa3. The means ± SD of 3 independent experiments are presented, relative to the siControl condition. The p values are shown (One sample t-test). **B.** Adhesions were analyzed in Fibronectin-plated HUVECs transfected with siControl or siRasa3 by confocal microscopy using an anti-Paxillin antibody. Histograms represent size distribution of paxillin positive adhesions in 35 control and 33 siRasa3-treated cells. Adhesions were classified into three size categories: (0.2–0.5 μm^2^), (0.5–1 μm^2^) and (>1 μm^2^). The p values are shown (Student’s t-test). **C.** Adhesions were analyzed in Fibronectin-plated HUVECs transfected with siControl and siRasa3 by confocal microscopy using an anti-Paxillin antibody (green). F-actin is visualized using Phalloidin (red). Representative images are shown. Bars = 10 μm. Quantification of the ratio of the length between the center and the mature focal adhesion (>1 μm^2^) versus the length between the center and the cell periphery (n = 244 and n = 343 for siCTL and siRasa3, respectively). The p value is shown (Student’s t-test). **D.** Adhesions were analyzed in VEGF-stimulated HUVECs transfected with siControl and siRasa3 as described in (**B**) in 21 control and 23 siRasa3-treated cells. The p values are shown (Student’s t-test).
(TIF)

S7 Fig Depletion of Rasa3 impairs activation of the FAK-Src complex. **A.** Detection of FAK, Src, Paxillin (Pax) and ERK phosphorylation levels in lysates from VEGF-stimulated, control and siRasa3-transfected HUVECs by Western blotting with phospho-specific antibodies. Total FAK, Src, Paxillin and ERK levels were respectively used as control. Phosphorylation levels of FAK, Src, Paxillin and ERK were quantified by densitometry as the ratio of phospho-specific signal over the total protein signal, relative to control HUVECs. Results are expressed as means ± SD from at least 3 independent experiments. The p values are shown (Student’s t-test). **B.** Immunofluorescence analysis of aortic ring sprouts from *R3*^f/f^ and *R3*^f/f^ iEC-Cre mice stained for the IB4 endothelial marker (lower) and with an anti-phospho-FAK antibody (upper). Representative images of minimum 5 sprouts per genotype in 3 independent experiments are shown. Bars = 50 μm. Quantification of anti-phosphoFAK mean fluorescence intensity (MFI) ± SEM (n = 5). The p value is shown (Unpaired t-test).
(TIF)

S8 Fig Rasa3 knockdown in HUVECs has no effect on active R-Ras level. The densitometric quantification of active R-Ras detected by Western blotting on protein extracts from siControl and siRasa3 HUVECs is expressed as means ± SD from 3 independent experiments. RLU: Relative Luminescence Unit. The p value is shown (Student’s t-test).
(TIF)

S9 Fig Depletion of Rasa3 stabilizes endothelial VE-Cadherin-based cell-cell junctions. **A.** Effect of Rasa3-silencing on endothelial cell junctions. Adherent junctions were analyzed in HUVECs transfected with siControl and siRasa3 by confocal microscopy using an anti-VE-cadherin antibody (green). Representative images are shown. Bars = 10 μm. (Right) The signal was quantified. Histograms are mean ratio of FAJ length versus the total junction length per cell. Results are from 30 cells. The p value is shown (Student’s t-test). **B.** VE-cadherin internalization was analyzed by confocal microscopy using an anti-VE-cadherin antibody (green) in control and Rasa3-depleted cells after an EGTA treatment (4 mM). Bars = 50μm **C.** Effect of Rasa3-silencing on endothelial permeability after an EGTA treatment (4 mM). Results are mean quantification of FITC-dextran ± SD from 4 independent experiments and relative to EGTA-treated control cells. The p values are shown (non-treated cells: Student’s t-test; EGTA-treated cells: One sample test). **D.** Detection of VE-cadherin and Src phosphorylation levels in total lysates from non-treated and EGTA-treated sicontrol and siRasa3-transfected cells by Western blotting with phospho-specific antibodies. Total VE-cadherin and Src levels respectively were used as control. Results are expressed as means ± SD from 4 independent experiments, relative to control HUVECs. The p values are shown (non-treated cells: One sample t-test; EGTA-treated cells: Student’s t-test).
(TIF)

S1 Table Endothelial specific or full deletion of exons 11–12 of the mouse *Rasa3* gene during embryonic life results in embryonic death. Newborns were genotyped 21 days after bird by PCR.
* Plugged *R3*^f/f^ females were ip injected with 5 mg of tamoxifen at E8.5, E9.5 and E10.5. Statistics (Student’s t-test): ***: P<0.001.
(DOCX)

S2 Table Antibodies used in the manuscript. (DOCX)

S1 Movie The ability of control HUVECs and HUVECs deficient for Rasa3 to form capillary-like networks was investigated *in vitro* by time-lapse microscopy. Silencing of Rasa3 expression using siRNA dramatically impaired formation of a continuous vascular-like network. Indeed, whereas Rasa3-deficient HUVECs initially formed branched networks, the branches were hypocellular and unstable leading to rapid collapse of the network.
(MP4)

S2 Movie Analysis of the dynamics of adhesion assembly and disassembly by TIRF microscopy on migrating GFP-paxillin positive HUVECs depleted or not for Rasa3. The analysis was focused on FAs maturing just below the lamella, which appeared both larger and longer lived in siRasa3 HUVECs than in siControl HUVECs.
(MP4)
