# Supplementary figures and images for: Rasa3 controls turnover of endothelial cell adhesion and vascular lumen integrity by a Rap1-dependent mechanism
Source: PLoS Genet. 2018 Jan 30;14(1):e1007195. doi: 10.1371/journal.pgen.1007195 (PMC5806903; doi:10.1371/journal.pgen.1007195)

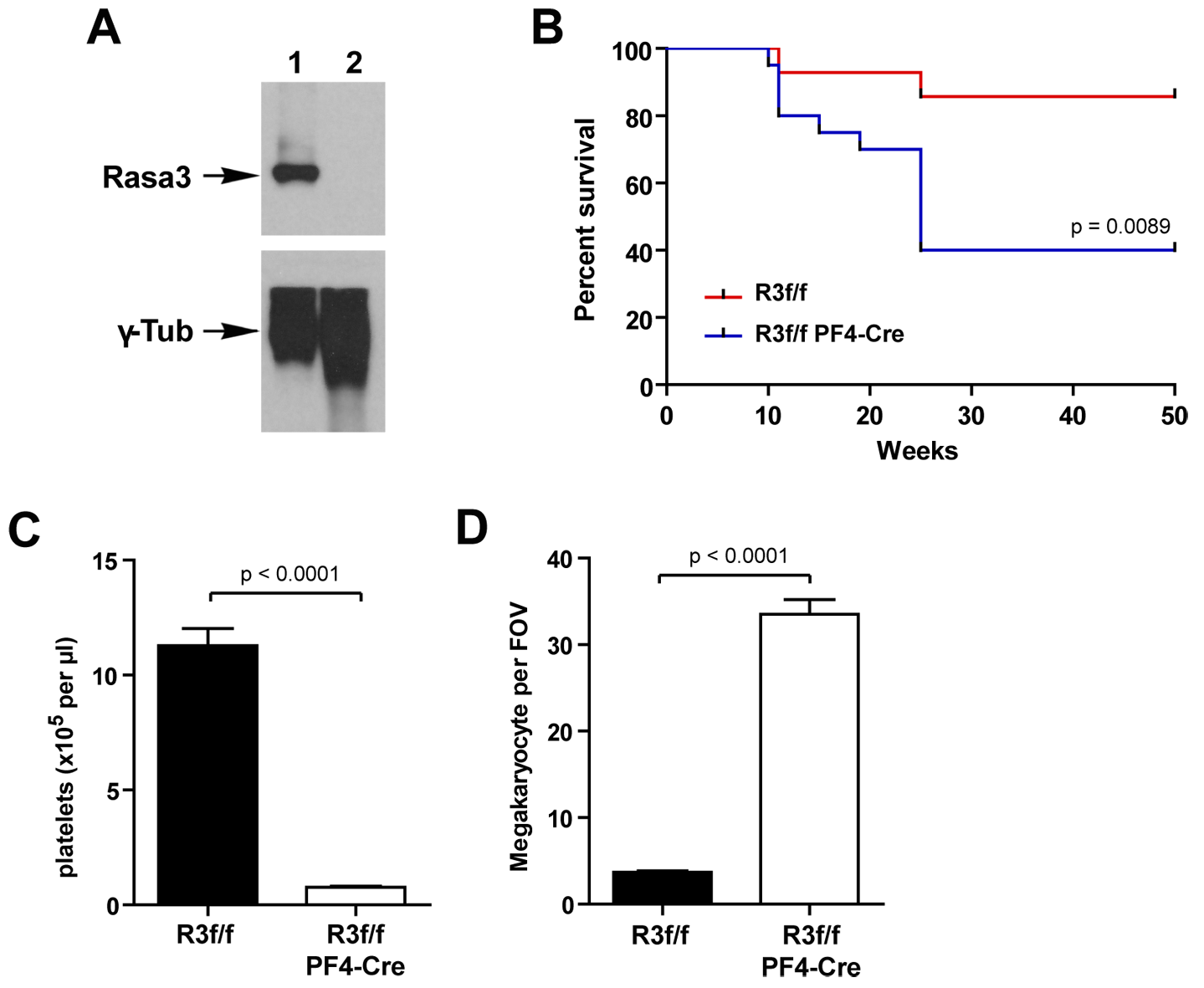

Supplement: S1 Fig — A. Immunodetection of Rasa3 and γ-Tubulin by Western blotting on washed-platelet extracts isolated from blood of R3f/f (1) and R3f/f PF4-Cre (2). Image is representative of 5 independent experiments. B. Survival curve of R3f/f (n = 11) and R3f/f PF4Cre (n = 8) mice over a period of 50 weeks. The p value is shown (Log-rank (Mantel-Cox) test). C. Blood platelet counts in adult R3f/f (n = 8) and R3f/f PF4-cre (n = 8) mice. Data are represented as mean ± SEM. The p value is shown (Unpaired t-test). D. Quantification of megakaryocytes present in the spleen of R3f/f (n = 6) and R3f/f PF4-Cre (n = 6) mice. Data are represented as mean ± SEM; the p value is shown (Unpaired t-test). (TIF) [file pgen.1007195.s001.tif]

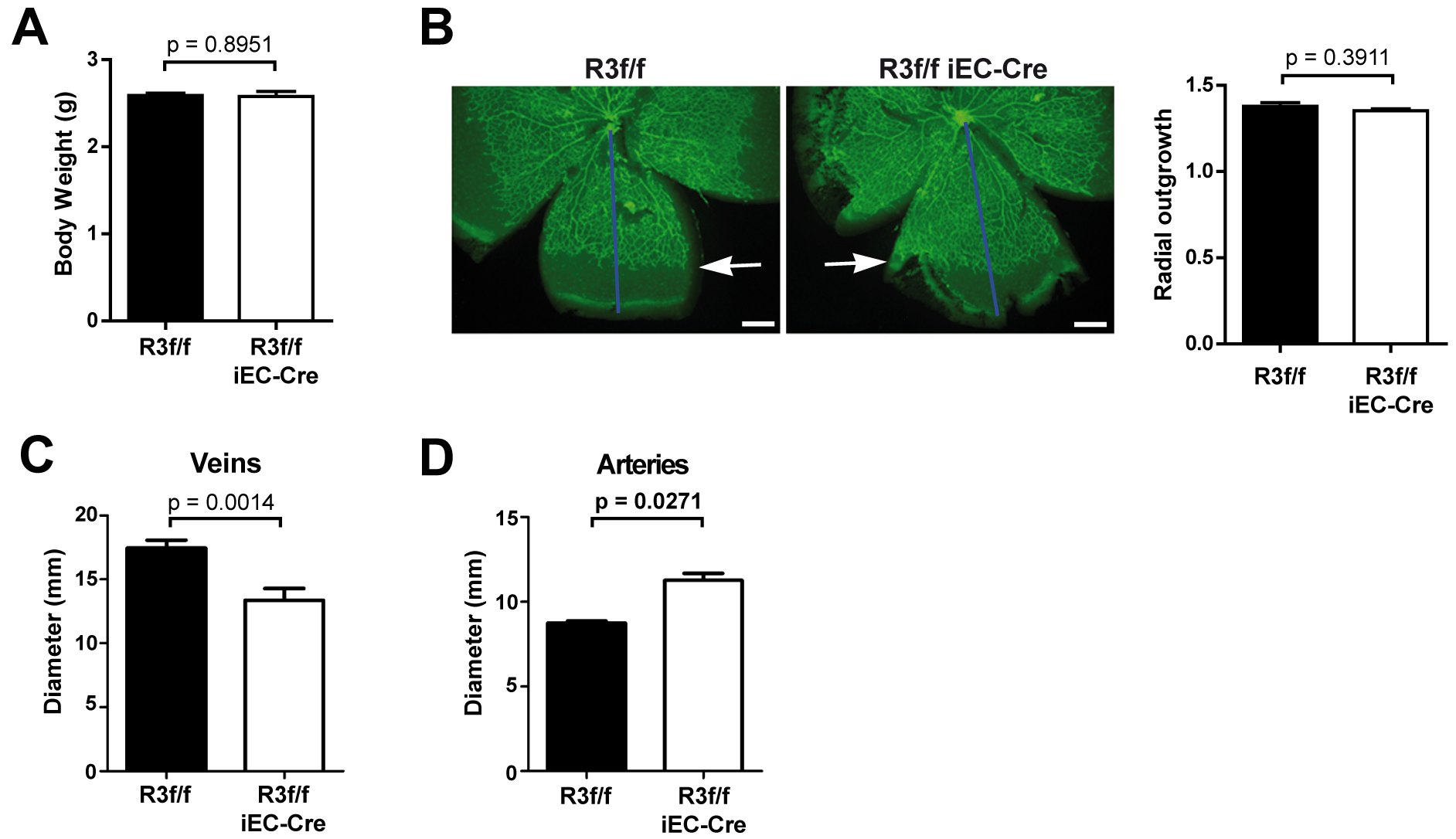

Supplement: S2 Fig — A. Quantification of R3f/f (n = 18) and R3f/f iEC-Cre (n = 8) newborn body weight at P5 after tamoxifen treatment. B. Representative image of R3f/f (n = 18) and R3f/f iEC-Cre (n = 8) newborn retina vasculature, stained with the IB4 (green) endothelial marker. The blue line and the white arrow indicate the total length and the vascular front of the vascular network, respectively. Bars = 2 mm. Quantification of the radial outgrowth of the retinal network is shown. C-D. Quantification of the vein (C) and arteries (D) diameter in P5 newborn retinas of R3f/f (n = 8) and R3f/f iEC-Cre (n = 8) newborns. Data are represented as mean ± SEM. The p values are shown (Unpaired t-test). (TIF) [file pgen.1007195.s002.tif]

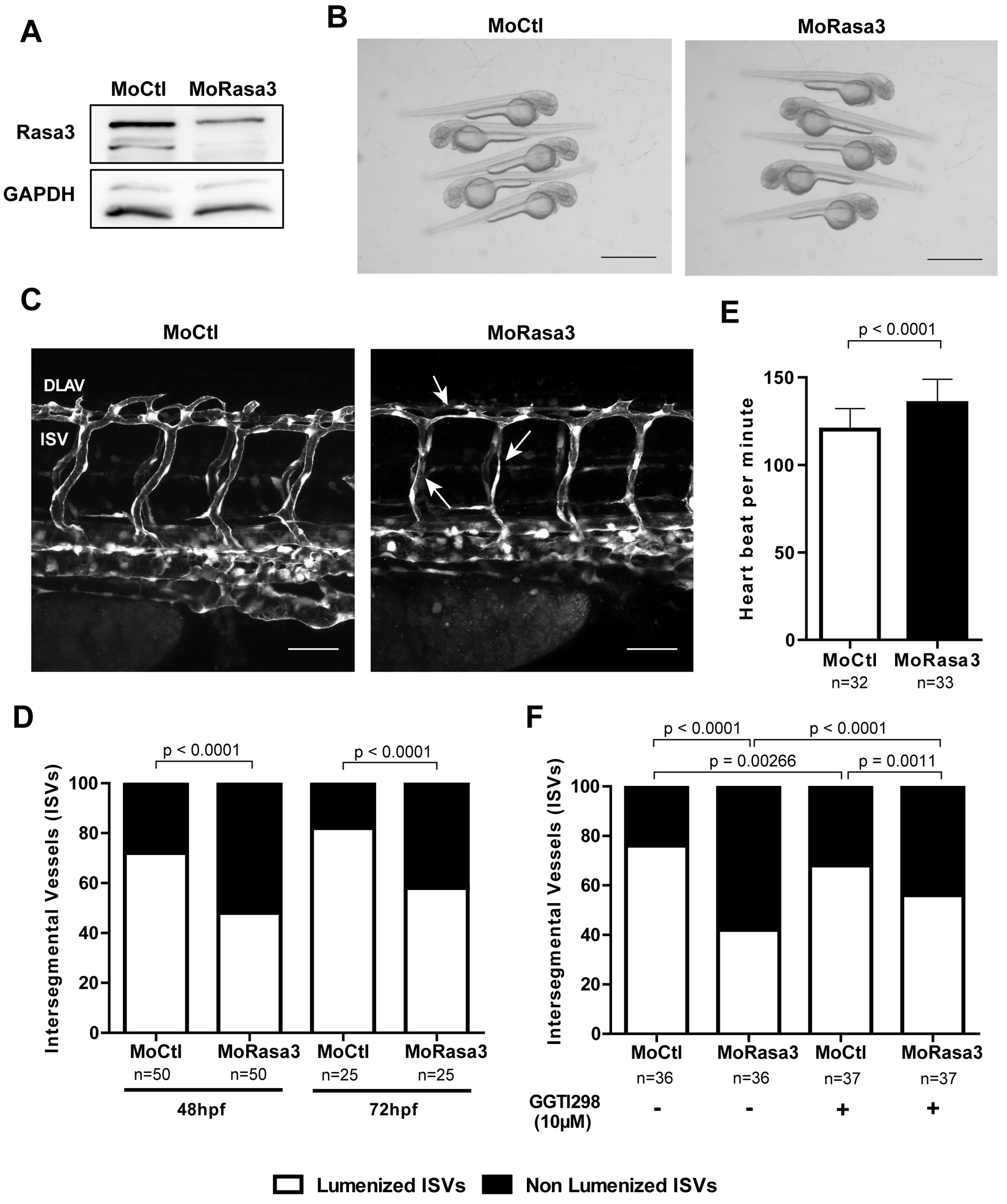

Supplement: S3 Fig — A. Detection of Rasa3 level in lysates from Control (Ctl) and Rasa morphant embryos. GAPDH was used as control. B. General morphology of Ctl and Rasa3 morphant embryos. C. Tg(fli1a:eGFP)y1 embryos were injected with control morpholino (MoCtl) or with morpholino targeting Rasa3 (MoRasa3). Confocal pictures of the trunk vasculature were taken at 48 hpf. Ctl embryos present normal ISVs and DLAVs with open lumen (arrow head) whereas Rasa3 morphant embryos show thinner, non-lumenized vessels (arrows). Bars = 50 mm. ISV, intersegmental vessel; DLAV, dorsal longitudinal anastomotic vessels. D. Quantification of lumenized ISVs in Ctl and Rasa3 morphant embryos at 48 and 72 hpf. The p values are shown (Fisher's exact test). Results are mean from 10 ISVs/embryo in 50 embryos at 48hpf and 25 embryos at 72hpf. E. Heart rates in 32 Ctl and 30 Rasa3 morphant embryos. Histograms are mean ± SD from 35 embryos. The p value is shown (Fisher's exact test). F. Rescue experiment using GGTI298 (10 μM). Quantification of lumenized ISVs in Ctl and Rasa3 morphant embryos at 48hpf. The p values are shown. Results are mean from 10 ISVs/embryo in 35 embryos. (TIF) [file pgen.1007195.s003.tif]

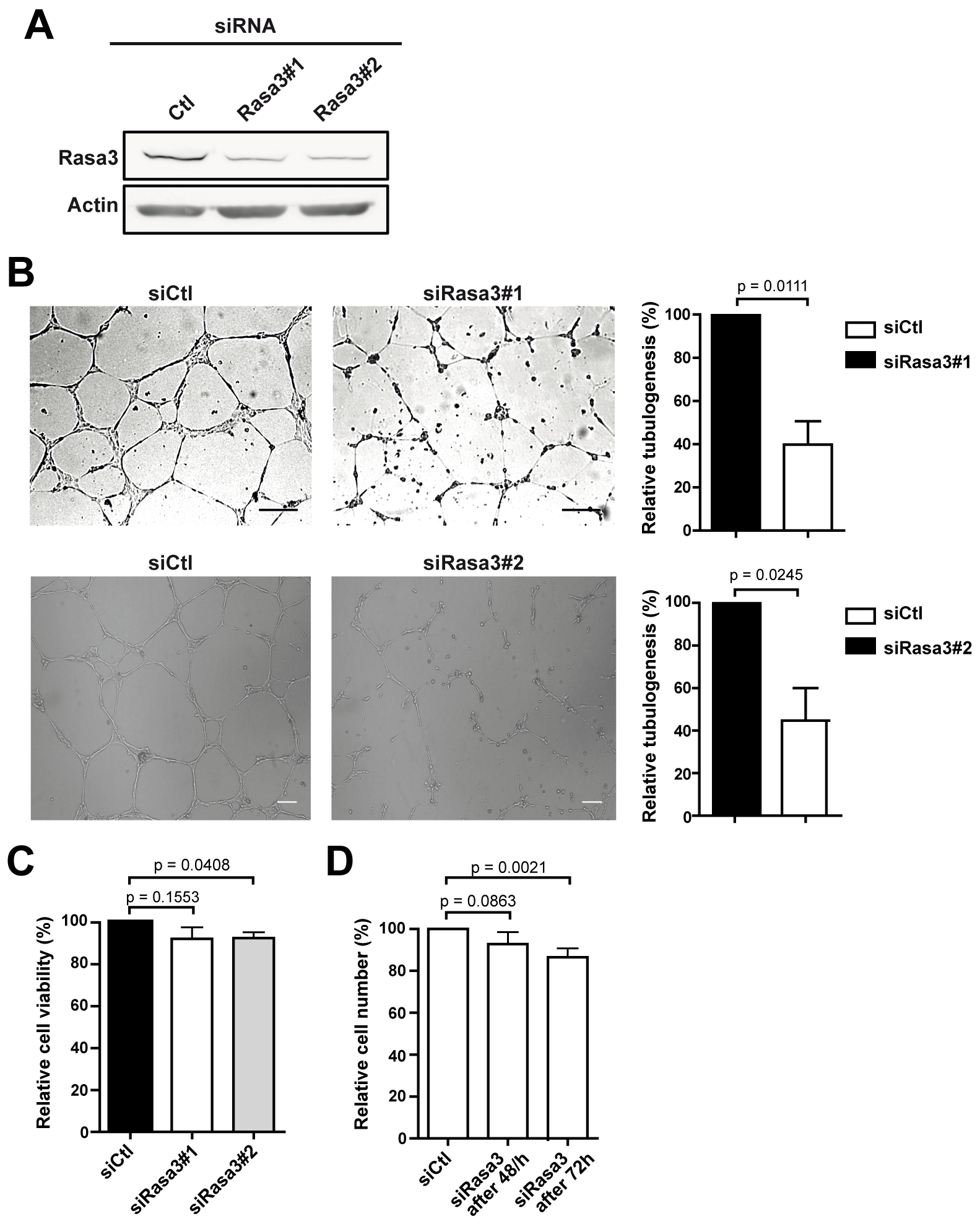

Supplement: S4 Fig — A. Immunodetection of Rasa3 and Actin by Western blotting on total extracts from HUVECs transfected with a control siRNA or two different Rasa3 siRNAs (siRasa3#1 and siRasa3#2). B. Representative micrographs of a tube-like formation assay in Matrigel using HUVECs treated with siControl or with two different Rasa-siRNA (siRasa3#1 and siRasa3#2). Images are representative of 3 independent experiments. Bar = 100 μm. Histograms represent mean ± SD of relative tubulogenesis of capillary-like structures measured in five different fields from 3 independent experiments. The p values are shown (One sample t-test). C. Histograms represent mean relative cell viability ± SD in siCTL or siRasa3-treated HUVECs from 3 independent experiments. D. Histograms represent mean cell number of siRasa3#1 at indicated time points after seeding and relative to the number of cells in siCTL-treated HUVECs. Results are from at least 3 independent experiments. The p values are shown in B, C and D (Student’s t-test). (TIF) [file pgen.1007195.s004.tif]

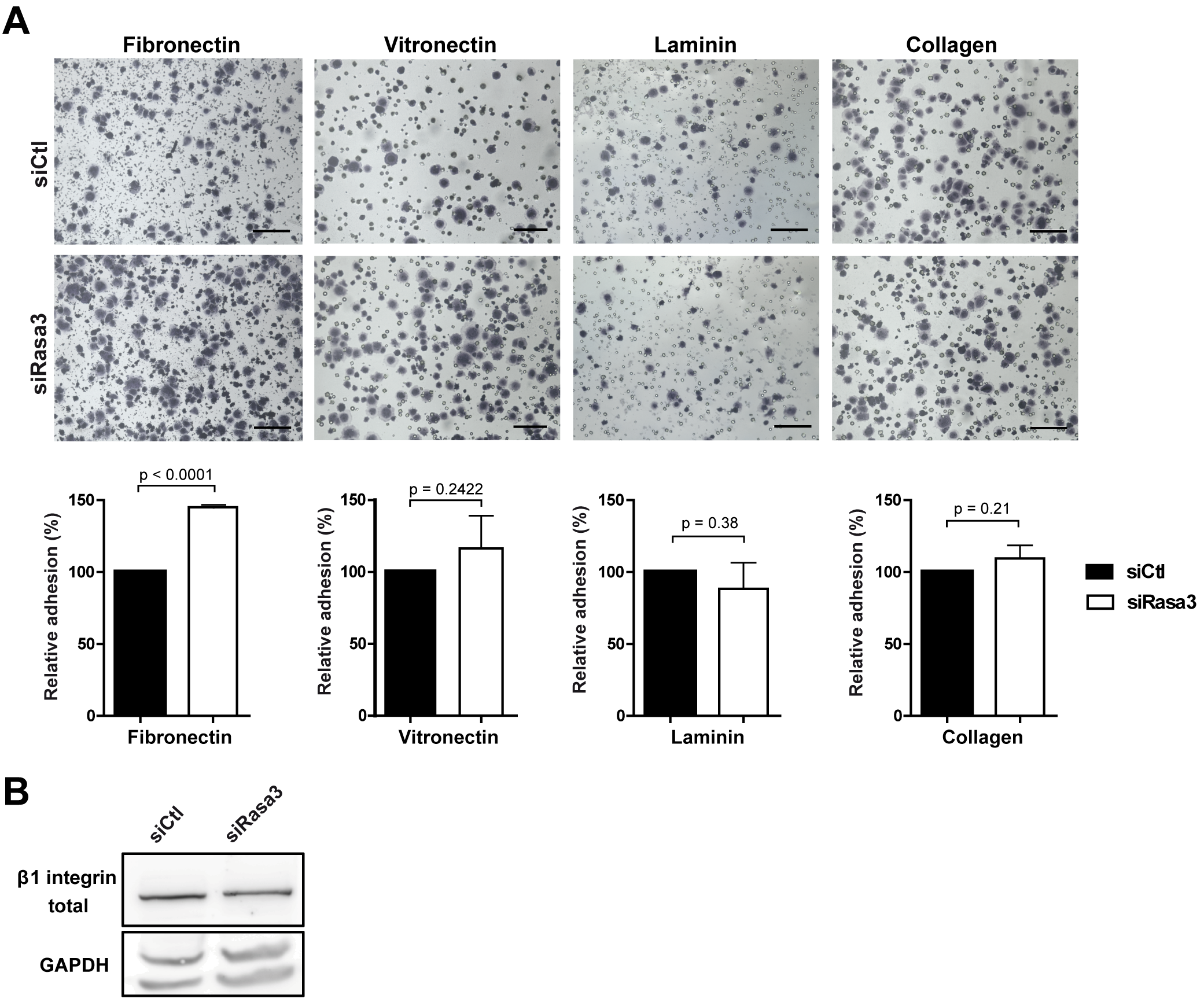

Supplement: S5 Fig — A. Effects of Rasa3-silencing on HUVEC adhesion onto Fibronectin, Collagen, Vitronectin or Laminin. Bars = 100 μm. Representative micrograph of an adhesion assay with HUVECs treated with siCTL or siRasa3. Images are representative from 3 to 5 independent experiments. Histograms are mean ± SD of 3 independent experiments. The p values are shown (Student’s t-test). B. Immunodetection of total integrin β1 levels by Western blotting on total extracts from HUVECs transfected with control or Rasa3 siRNA (related to the experiment described in Fig 5A). Actin was used as a loading control. (TIF) [file pgen.1007195.s005.tif]

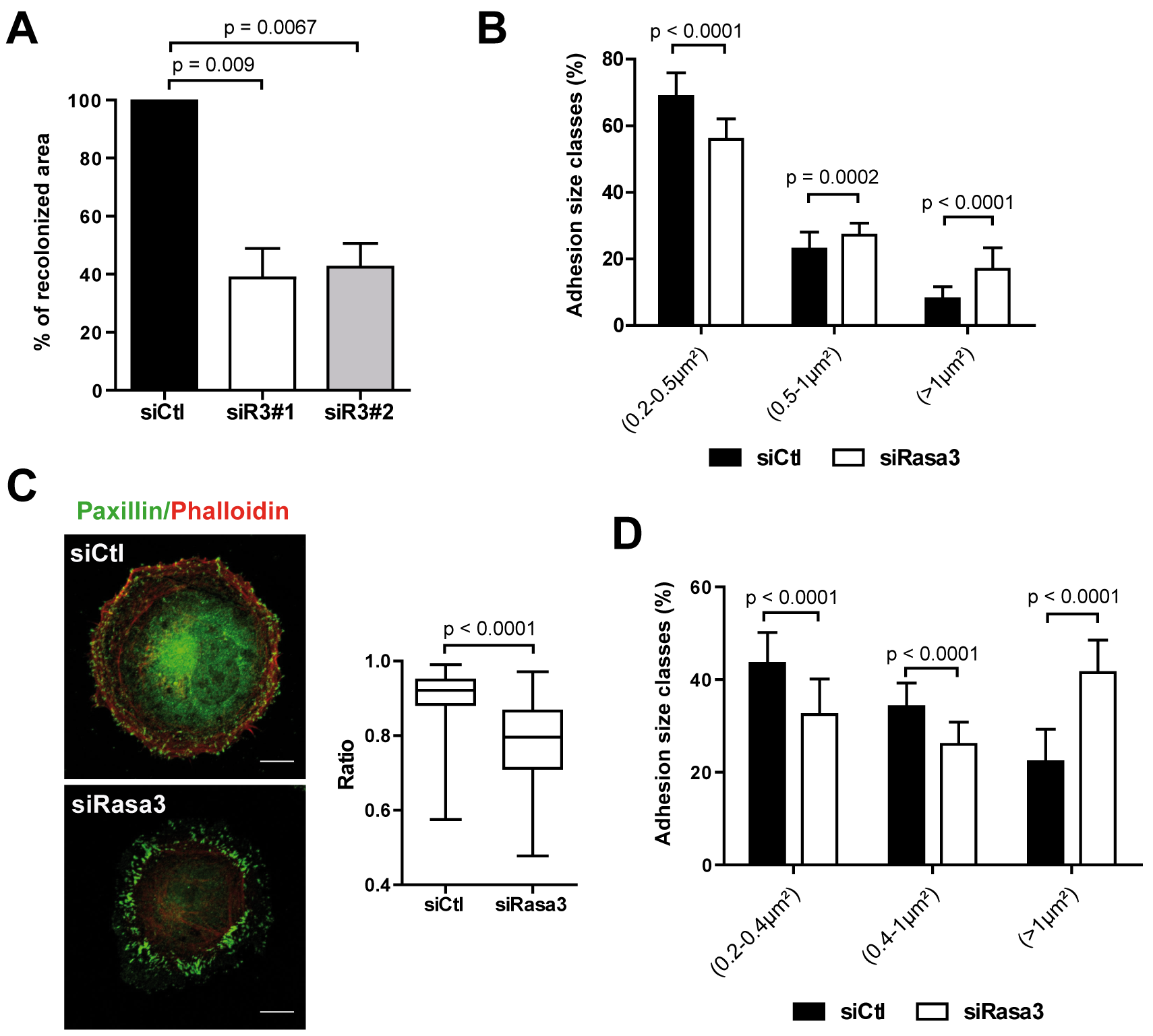

Supplement: S6 Fig — A. In a scratch-wound migration assay, the recolonized area was analyzed at 5h in HUVECs transfected with siControl or two different siRasa3. The means ± SD of 3 independent experiments are presented, relative to the siControl condition. The p values are shown (One sample t-test). B. Adhesions were analyzed in Fibronectin-plated HUVECs transfected with siControl or siRasa3 by confocal microscopy using an anti-Paxillin antibody. Histograms represent size distribution of paxillin positive adhesions in 35 control and 33 siRasa3-treated cells. Adhesions were classified into three size categories: (0.2–0.5 μm2), (0.5–1 μm2) and (>1 μm2). The p values are shown (Student’s t-test). C. Adhesions were analyzed in Fibronectin-plated HUVECs transfected with siControl and siRasa3 by confocal microscopy using an anti-Paxillin antibody (green). F-actin is visualized using Phalloidin (red). Representative images are shown. Bars = 10 μm. Quantification of the ratio of the length between the center and the mature focal adhesion (>1 μm2) versus the length between the center and the cell periphery (n = 244 and n = 343 for siCTL and siRasa3, respectively). The p value is shown (Student’s t-test). D. Adhesions were analyzed in VEGF-stimulated HUVECs transfected with siControl and siRasa3 as described in (B) in 21 control and 23 siRasa3-treated cells. The p values are shown (Student’s t-test). (TIF) [file pgen.1007195.s006.tif]

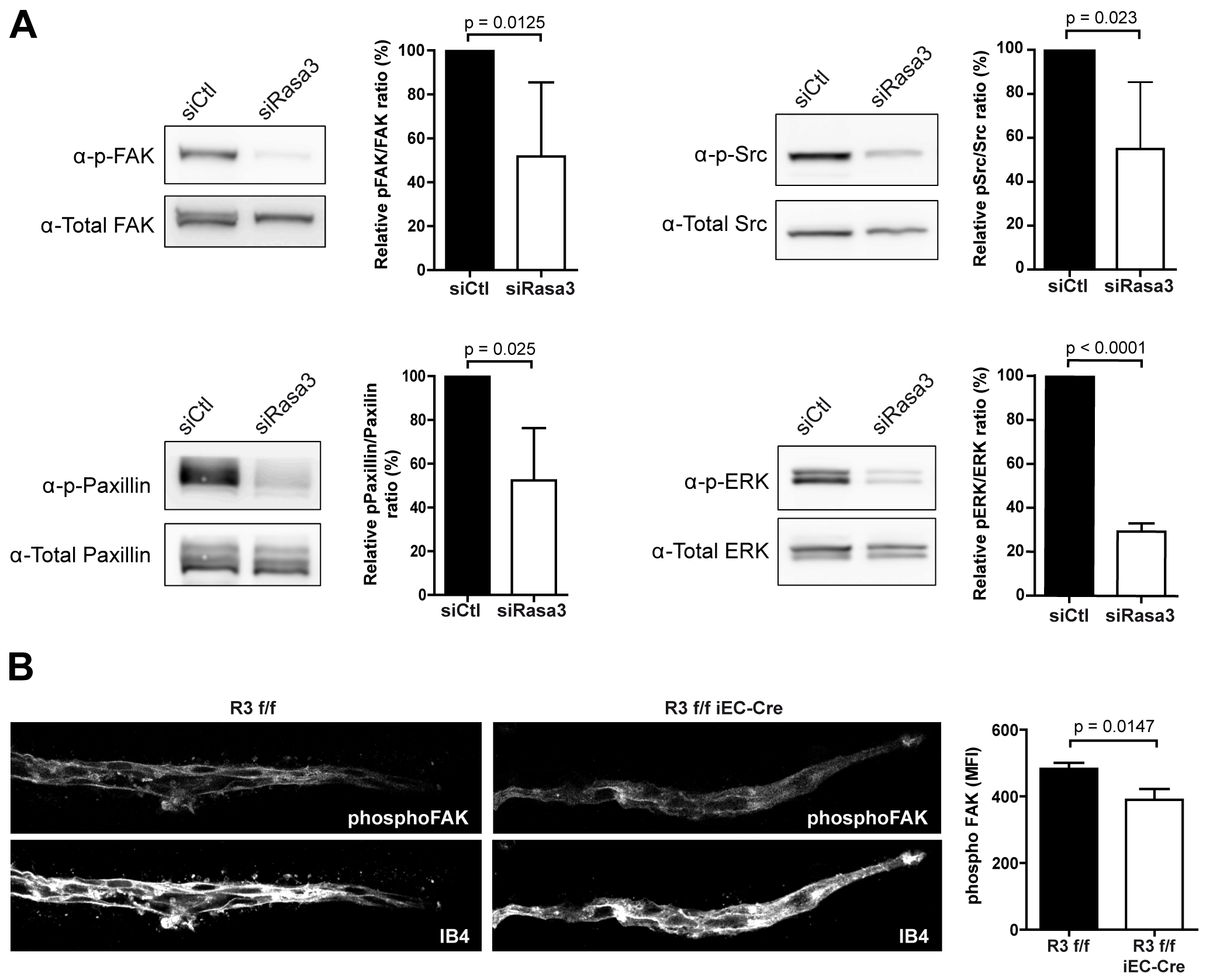

Supplement: S7 Fig — A. Detection of FAK, Src, Paxillin (Pax) and ERK phosphorylation levels in lysates from VEGF-stimulated, control and siRasa3-transfected HUVECs by Western blotting with phospho-specific antibodies. Total FAK, Src, Paxillin and ERK levels were respectively used as control. Phosphorylation levels of FAK, Src, Paxillin and ERK were quantified by densitometry as the ratio of phospho-specific signal over the total protein signal, relative to control HUVECs. Results are expressed as means ± SD from at least 3 independent experiments. The p values are shown (Student’s t-test). B. Immunofluorescence analysis of aortic ring sprouts from R3f/f and R3f/f iEC-Cre mice stained for the IB4 endothelial marker (lower) and with an anti-phospho-FAK antibody (upper). Representative images of minimum 5 sprouts per genotype in 3 independent experiments are shown. Bars = 50 μm. Quantification of anti-phosphoFAK mean fluorescence intensity (MFI) ± SEM (n = 5). The p value is shown (Unpaired t-test). (TIF) [file pgen.1007195.s007.tif]

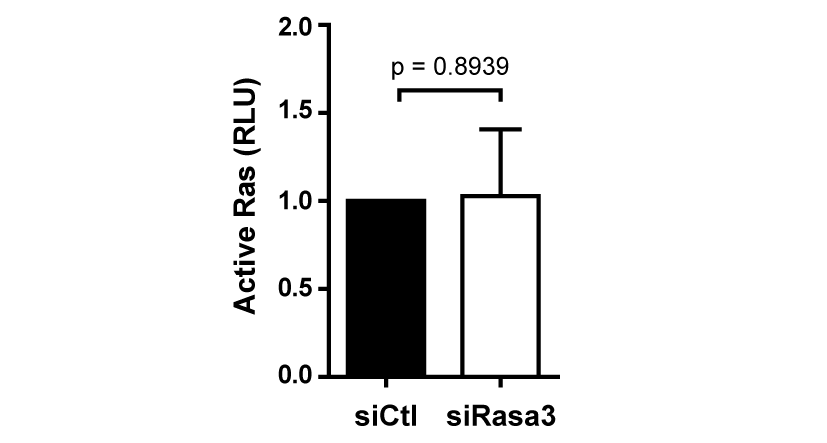

Supplement: S8 Fig — The densitometric quantification of active R-Ras detected by Western blotting on protein extracts from siControl and siRasa3 HUVECs is expressed as means ± SD from 3 independent experiments. RLU: Relative Luminescence Unit. The p value is shown (Student’s t-test). (TIF) [file pgen.1007195.s008.tif]

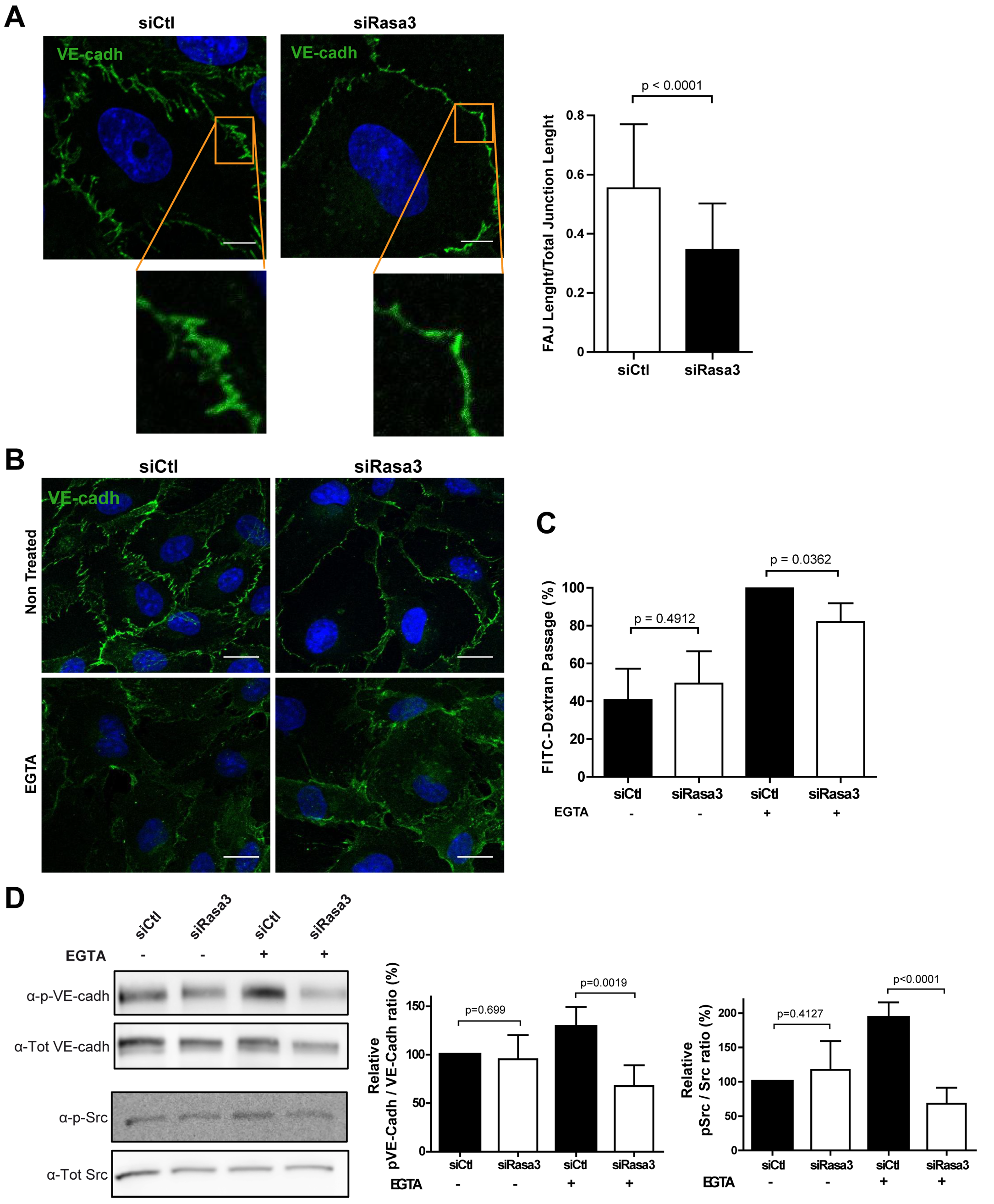

Supplement: S9 Fig — A. Effect of Rasa3-silencing on endothelial cell junctions. Adherent junctions were analyzed in HUVECs transfected with siControl and siRasa3 by confocal microscopy using an anti-VE-cadherin antibody (green). Representative images are shown. Bars = 10 μm. (Right) The signal was quantified. Histograms are mean ratio of FAJ length versus the total junction length per cell. Results are from 30 cells. The p value is shown (Student’s t-test). B. VE-cadherin internalization was analyzed by confocal microscopy using an anti-VE-cadherin antibody (green) in control and Rasa3-depleted cells after an EGTA treatment (4 mM). Bars = 50μm C. Effect of Rasa3-silencing on endothelial permeability after an EGTA treatment (4 mM). Results are mean quantification of FITC-dextran ± SD from 4 independent experiments and relative to EGTA-treated control cells. The p values are shown (non-treated cells: Student’s t-test; EGTA-treated cells: One sample test). D. Detection of VE-cadherin and Src phosphorylation levels in total lysates from non-treated and EGTA-treated sicontrol and siRasa3-transfected cells by Western blotting with phospho-specific antibodies. Total VE-cadherin and Src levels respectively were used as control. Results are expressed as means ± SD from 4 independent experiments, relative to control HUVECs. The p values are shown (non-treated cells: One sample t-test; EGTA-treated cells: Student’s t-test). (TIF) [file pgen.1007195.s009.tif]
