# Supplementary material for: Rasa3 controls turnover of endothelial cell adhesion and vascular lumen integrity by a Rap1-dependent mechanism
Source: PLoS Genet. 2018 Jan 30;14(1):e1007195. doi: 10.1371/journal.pgen.1007195 (PMC5806903; doi:10.1371/journal.pgen.1007195)
Supplement: S1 Table — Newborns were genotyped 21 days after bird by PCR. * Plugged R3f/f females were ip injected with 5 mg of tamoxifen at E8.5, E9.5 and E10.5. Statistics (Student’s t-test): ***: P<0.001. (DOCX) [file pgen.1007195.s010.docx]

**S1 Table: Endothelial specific or full deletion of exons 11-12 of the mouse *Rasa3* gene during embryonic life results in embryonic death.** Newborns were genotyped 21 days after bird by PCR.

| **Couple (♂ x ♀):** | **Newborns genotype** | **Observed number (%)** | **Expected %** |
| --- | --- | --- | --- |
| *R3*^∆/+^ x *R3*^∆/+^ | ***R3*^+/+^** | 36/115 (**31.3**) | 25 |
|  | ***R3*^∆/+^** | 79/115 (**68.7**) | 50 |
|  | ***R3*^∆/∆^** | 0/115 (**0**)*** | 25 |
| *R3*^f/+^ PF4-Cre x *R3*^f/f^ | ***R3*^f/f^** | 21/98 (**21.43**) | 25 |
|  | ***R3*^f/+^** | 27/98 (**27.55**) | 25 |
|  | ***R3*^f/+^ PF4-Cre** | 26/98 (**26.53**) | 25 |
|  | ***R3*^f/f^ PF4-Cre** | 24/98 (**24.48**) | 25 |
| *R3*^f/f^ iEC-Cre x *R3*^f/f^ ***** | ***R3*^f/f^** | 41/41 (**100**) | 50 |
|  | ***R3*^f/f^ iEC-Cre** | 0/41 (**0**)*** | 50 |

* Plugged *R3*^f/f^ females were ip injected with 5 mg of tamoxifen at E8.5, E9.5 and E10.5. Statistics (Student’s t-test): ***: P<0.001.
