# Supplementary material for: Rasa3 controls turnover of endothelial cell adhesion and vascular lumen integrity by a Rap1-dependent mechanism
Source: PLoS Genet. 2018 Jan 30;14(1):e1007195. doi: 10.1371/journal.pgen.1007195 (PMC5806903; doi:10.1371/journal.pgen.1007195)
Supplement: S2 Table — (DOCX) [file pgen.1007195.s011.docx]

**S2 Table: Antibodies used in the manuscript.**

| In vitro | | |
| --- | --- | --- |
| **Antibody** | **Company** | **Reference** |
| Rasa3 | Santa Cruz | sc-166442 |
| Paxillin | BD BioSciences | 610051 |
| Phospho-paxillin | Cell Signaling | 2541S |
| Rap1 | Cell Signaling | 8825S |
| FAK | Abcam | ab72140 |
| phosphoFAK | Thermo-fisher | 44-624G |
| ERK | Cell Signaling | 9102s |
| PhosphoERK | Cell Signaling | 9101s |
| SRC | Cell Signaling | 2123 |
| PhosphoSRC | Cell Signaling | 2101S |
| VE-cadherin | Santa Cruz | sc-9989 |
| Phospho- VEcadherin | Invitrogen | 44-1144G |
| B1 integrin | EMD Millipore | MAB-1965 |
| Activated B1 integrin | BD Pharmigen | 550531 |
| GAPDH | SantaCruz | sc-166545 |
| pMLC2 | Cell Signaling | 3674 |
| Alpha-tubulin | Sigma | T6199 |
| Acetyl alpha-tubulin | EMD Millipore | MABT868 |
| Phalloidin-568 | Thermofisher | A12380 |
| Ras | Cell Signaling | 8832S |
|  | | |
| In vivo and ex vivo | | |
| **Antibody** | **Company** | **Ref** |
| Rasa3 | Abnova | H00022821-D01P |
| IB4-Biotin | Sigma | L2140 |
| NG2 | EMD Millipore | AB5320 |
| Activated B1 integrin | BD Pharmigen | 550531 |
| active Rap1 | New East Bioscience | 26912 |
| phosphoFAK | Cell Signaling | 3283 |
| Phalloidin-TRICT | Sigma | P1951 |
| Gamma-tubulin | Sigma | T6557 |
| Acetyl alpha-tubulin | Abcam | ab24610 |
|  | | |
| Conjugated secondary antibodies | | |
| Anti-mouse-IgG 488 | Sigma | 62197 |
| Anti-Rabbit- IgG 488 | Sigma | 18772 |
| Anti-mouse- IgG 647 | Sigma | 50185 |
| Anti-Rabbit- IgG 647 | Sigma | 40839 |
| Anti-mouse- IgG 594 | Sigma | 76085 |
| Anti-Rabbit- IgG 594 | Sigma | 77671 |
| Streptavidin- 647 | Molecular Probes | S32357 |
| Streptavidin- 488 | Molecular Probes | S32354 |
| Streptavidin- 594 | Molecular Probes | S32356 |
| Anti-Rabbit-Alexa Pacific Blue | Molecular Probes | P-10994 |
